# Public transcriptome database-based selection and validation of reliable reference genes for breast cancer research

**DOI:** 10.1186/s12938-021-00963-8

**Published:** 2021-12-11

**Authors:** Qiang Song, Lu Dou, Wenjin Zhang, Yang Peng, Man Huang, Mengyuan Wang

**Affiliations:** 1 Department of Central Laboratory, Chongqing University Three Gorges Hospital, School of Medicine, Chongqing University, Chongqing, 404000 China; 2 Department of Endocrine and Breast Surgery, The First Affiliated Hospital of Chongqing Medical University, Chongqing, 400016 China; 3 Department of Breast Surgery, Chongqing University Three Gorges Hospital, No.165, Xin Cheng Lu, Wanzhou, Chongqing, 404000 China

**Keywords:** Reference genes, Breast cancer, qRT-PCR, Normalization, Gene expression

## Abstract

**Background:**

Quantitative reverse transcription-polymerase chain reaction (qRT-PCR) is the most sensitive technique for evaluating gene expression levels. Choosing appropriate reference genes (RGs) is critical for normalizing and evaluating changes in the expression of target genes. However, uniform and reliable RGs for breast cancer research have not been identified, limiting the value of target gene expression studies. Here, we aimed to identify reliable and accurate RGs for breast cancer tissues and cell lines using the RNA-seq dataset.

**Methods:**

First, we compiled the transcriptome profiling data from the TCGA database involving 1217 samples to identify novel RGs. Next, ten genes with relatively stable expression levels were chosen as novel candidate RGs, together with six conventional RGs. To determine and validate the optimal RGs we performed qRT-PCR experiments on 87 samples from 11 types of surgically excised breast tumor specimens (*n* = 66) and seven breast cancer cell lines (*n* = 21). Five publicly available algorithms (geNorm, NormFinder, ΔCt method, BestKeeper, and ComprFinder) were used to assess the expression stability of each RG across all breast cancer tissues and cell lines.

**Results:**

Our results show that RG combinations *SF1* + *TRA2B* + *THRAP3* and *THRAP3* + *RHOA* + *QRICH1* showed stable expression in breast cancer tissues and cell lines, respectively, and that they displayed good interchangeability. We propose that these combinations are optimal triplet RGs for breast cancer research.

**Conclusions:**

In summary, we identified novel and reliable RG combinations for breast cancer research based on a public RNA-seq dataset. Our results lay a solid foundation for the accurate normalization of qRT-PCR results across different breast cancer tissues and cells.

**Supplementary Information:**

The online version contains supplementary material available at 10.1186/s12938-021-00963-8.

## Introduction

Quantitative reverse transcription-polymerase chain reaction (qRT-PCR) is a highly sensitive and low-cost technique that is widely used in molecular biology [1]. However, the accuracy and interpretation of its results are determined by the stability of the selected reference genes (RGs) [2]. Hence, the selection of suitable RGs is the first aim of any research system dedicated to the investigation of differential gene expression [3]. Furthermore, the simultaneous use of multiple RGs will result in more accurate data on target gene expression [2, 4].

### Related works

Breast cancer is the most common malignancy in females and accounts for approximately 30% of all cancers diagnosed [5]. Based on the expression of hormone receptors (HR), including the estrogen receptor (ER), progesterone receptor (PR), and the human epidermal growth factor receptor 2 (HER-2), breast cancer can be classified into four subtypes including HR + HER2 −, HR + HER2 + , HR-HER2 + , and HR-HER2 − [6]. During the course of breast cancer treatment, subtype status determines the use of neoadjuvant chemotherapy (NAC). In addition, breast disease also includes benign tumors [7]. Tumorigenesis and breast cancer metastasis are associated with gene expression changes that are most commonly detected using qRT-PCR [8]. In previous breast cancer studies, commonly used RGs included beta-actin (ACTB), glyceraldehyde-3-phosphate dehydrogenase (GAPDH), beta-glucuronidase (GUSB), ribosomal protein L13a (RPL13A), and tubulin alpha 1a (TUBA1A) [3, 9]. However, research has indicated that these RGs are not consistently expressed across different tissues and experimental conditions [8, 10, 11]. Therefore, it is crucial to identify new RGs whose expression across various breast cancer tissues is more consistent.

Many novel RGs have been predicted and validated in many species and disease models, such as traumatic brain injury [12], *Euscaphis konishii Hayata* [13], *Salix viminalis* [4], *Oryza sativa* [14], *Gentiana macrophylla* [15], *Homo sapiens* [16], and *Rhizophora apiculate* [17]. However, to our knowledge, few systematic studies have been conducted to validate potential RGs for breast cancer. Available studies involved either tissues or cell lines (but not both), and the RGs concerned were not novel [3, 8, 18–20]. Considering the enormous threat breast cancer poses to human health, the identification of RGs that are more relevant to a wide range of breast cancer tissues and cells across several conditions is urgently needed [21–23]. In this work, we hypothesized that novel RGs for breast cancer research could be identified and validated using an mRNA-seq dataset.

### Contributions

To this end, we employed the mRNA-seq datasets from The Cancer Genome Atlas (TCGA) to discover novel RGs. Ten genes that displayed a relatively stable expression (*SF1*, *TARDBP*, *THRAP3*, *QRICH1*, *TRA2B*, *SRSF3*, *YY1*, *DNAJC8*, *RNF10,* and *RHOA*) and six traditional RGs (*ACTB*, *TUBA1A*, *RPL13A*, *B2M*, *GAPDH,* and *GUSB*) were selected as the candidate RGs. The qRT-PCR experiments were performed on different experimental samples including 11 types of breast cancer tissues and seven different breast cancer cell lines. The stability of expression of these candidate RGs was calculated using geNorm [24], NormFinder [25], ΔCtmethod [26], BestKeeper [27], and ComprFinder [28]. Finally, the optimal RGs were validated and confirmed. Our study significantly improves upon previous work in breast cancer research.

## Results

### Identification of candidate RGs based on a public transcriptomic database

**Fig. 1:**
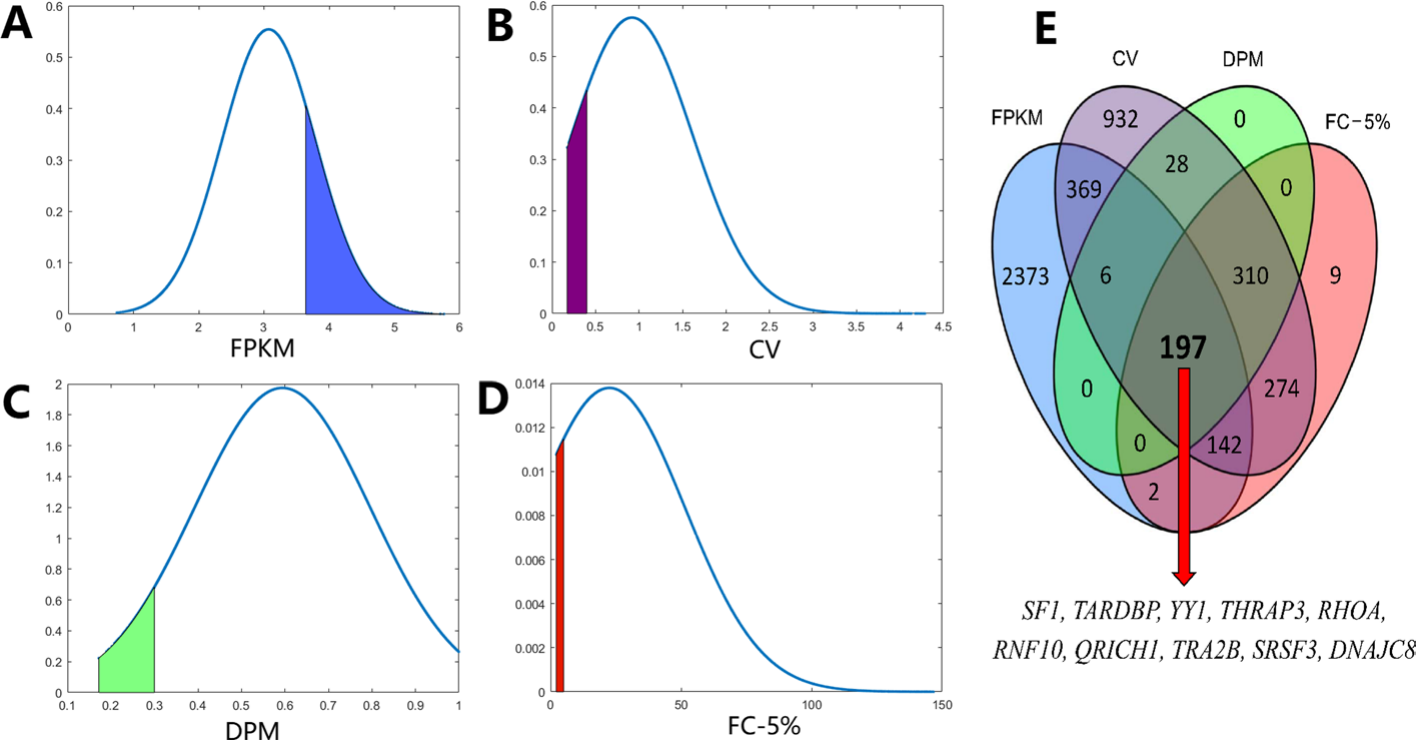
Probability density curve of FPKM, CV, DPM, and FC-5% in 15,458 unigenes. The y-axes indicate the probability values for all 15,457 genes. The candidate reference genes (RGs) were preliminarily screened by four criteria including FPKM ≥ 10, CV ≤ 0.4, DPM ≤ 0.3, and FC-5% ≤ 4 (**A–D**). The 197 RGs that met various criteria were identified by Venn diagram analysis (**E**), and among these, 10 genes were selected as the novel candidate RGs. FPKM, gene fragments per kilobase of exon model per million mapped reads; CV, coefficient of variation; DPM, dispersion measure; FC-5%, the fold change between the top 5% highest expression levels divided by the bottom 5% within the 1217 samples

Transcriptome sequencing data of 1217 breast cancer samples were obtained from the TCGA database. Next, 15,450 unigenes that were identified after processing were evaluated by Fragments Per Kilobase Million (FPKM) (high expression level, FPKM ≥ 10), coefficients of variation (CV) (low variability as determined by CV ≤ 40%), fold change (FC)-5% (the top 5% of 1217 samples divided by the lowest 5%, FC-5% ≤ 5), and dispersion measure (DPM) (DPM ≤ 0.3) values. The results for the different statistical algorithms, shown in Fig. 1, were as follows:

1. FPKM: A total of 4723 genes satisfied the requirement (30.57% of 15,450, the blue area in Fig. 1A).
2. CV (%): There were 2649 genes with a CV ≤ 40% (17.15% of 15,450, the purple area in Fig. 1B) after filtering.
3. FC-5%: This parameter allowed the identification of 2287 genes (14.80% of 15,450, the green area in Fig. 1C).
4. DPM: This parameter resulted in the identification of 464 genes (3.00% of 15,450, the red area in Fig. 1D).

Gene overlap between the four algorithms was identified using a Venn diagram with 4-color blocks (blue, purple, green, and red), showing that 197 genes satisfied all four requirements (Fig. 1E). Of these 197 genes, 10 genes (*SF1*, *TARDBP*, *THRAP3*, *QRICH1*, *TRA2B*, *SRSF3*, *YY1*, *DNAJC8*, *RNF10,* and *RHOA*) were selected as novel candidate RGs due to their higher FPKM values and easier primers design. In addition, *GUSB*, *TUBA1A*, *RPL13A,* and *B2M*, which previous studies suggested being stable RGs in breast cancer research, and two classical RGs, *ACTB* and *GAPDH*, were also considered. These genes were ranked based on their CV values (shown in Table 1).

**Table 1:** The summarized information of 16 potential RGs based on transcriptome data

Gene	FPKM	CV	FC-5%	DPM	Order
*SF1*	38.65	21.70%	2.52	0.21	5
*TARDBP*	20.30	18.51%	2.32	0.18	1
*THRAP3*	41.96	24.44%	3.10	0.24	16
*QRICH1*	14.67	25.76%	3.11	0.25	45
*TRA2B*	11.66	24.14%	2.78	0.23	12
*SRSF3*	39.09	24.08%	2.76	0.23	11
*YY1*	15.20	24.75%	2.83	0.24	23
*DNAJC8*	30.96	24.59%	2.92	0.24	19
*RNF10*	32.58	24.62%	2.78	0.24	21
*RHOA*	223.73	25.60%	3.05	0.25	40
*ACTB*	1490.51	38.06%	5.02	0.36	1834
*TUBA1A*	72.98	59.42%	12.88	0.51	6728
*RPL13A*	716.37	56.46%	8.78	0.49	6189
*B2M*	625.30	64.66%	12.56	0.54	7483
*GAPDH*	739.50	72.26%	11.79	0.59	8454
*GUSB*	31.47	142.43%	11.33	0.82	12,695

### Primer specificity and amplification efficiency for qRT-PCR

A total of 20 paired primers including 16 candidate RGs and 4 target genes were designed for qRT-PCR experiments. The unigene name, ENSid, gene description, primer sequences, theoretical Tm (°C), product size, primer efficiency (E), and coefficient of determination (*R*^2^) are summarized in Table S1. The primer efficiency for all 20 genes ranged from 90.0% for *YY1* to 105.4% for *DNAJC8*, and correlation coefficients varied from 0.996 (*ACTB*) to 0.999 (*B2M*, *YY1*). All paired primers showed adequate specificity (Additional file 1: Fig. S1).

### Ct values of candidate reference genes

**Fig. 2:**
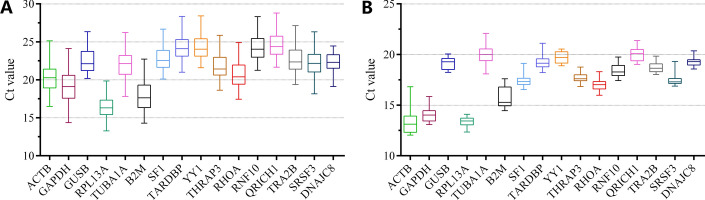
Distribution of Ct values for 16 candidate reference genes. Boxplot of absolute Ct value of the 16 candidate genes in breast cancer tissue samples (**A**) and cell lines (**B**). Boxes indicated median (Q2) and first and third quartiles (Q1 and Q3, respectively), and whiskers corresponded to the minimum and maximum values. The flatter the box, the more stable the gene expression

The mean Ct values (average of 3 technical replicates) for all 16 RGs are shown in Fig. 2 and Additional file 4: Table S2. The Ct values varied from 16.35 (*RPL13A*) to 24.57 (*QRICH1*) across various breast cancer tissues (Fig. 2A). The top 3 genes with low standard deviations were *DNAJC8* (1.17), *RPL13A* (1.36), and *SF1* (1.51). The 3 most differentially expressed genes were *GAPDH* (2.03), *B2M* (1.93), and *ACTB* (1.91). However, the Ct values of the breast cancer cell lines were overall lower than those of breast cancer tissues (Fig. 2B). A similar result of standard deviations was obtained in the breast cancer cells. To estimate the gene expression stability of these candidate RGs, more scientific algorithms will have to be introduced and used.

### Expression stability of candidate reference genes

In this study, the qRT-PCR data matrix was analyzed using five differential algorithms: geNorm, NormFinder, BestKeeper, ΔCtmethod, and ComprFinder.

#### geNorm analysis

Gene expression stability was evaluated by the M value using geNorm analysis. This program determines the pairwise variation of each gene with all other analyzed genes under the same experimental conditions: the lower the M value, the more stable the gene expression. In the breast cancer tissue group, the three most stably expressed genes (with the lowest M values) were *SF1*, *THRAP3*, and *TARDBP,* while *GAPDH*, *DNAJC8*, and *B2M* were the least stably expressed genes (Table 2). In the breast cancer cell group *THRAP3*, *RHOA*, and *QRICH1* were the top three stably expressed genes, while *B2M*, *TUBA1A*, and *ACTB* were the least stably expressed genes (Table 3). Among all samples, *TARDBP* was the most stably expressed gene, followed by *SF1* and *QRICH1*. Conversely, *TUBA1A*, *B2M*, and *ACTB* were the least stably expressed RGs (Additional file 5: Table S3).

**Table 2:** Gene expression stability calculated by 5 algorithms in all BC tissue samples

Gene	geNorm	NormFinder	BestKeeper	ΔCt method	ComprFinder
*SF1*	0.369(1)	0.018(5)	1.233(4)	0.626(4)	0.146(1)
*TRA2B*	0.455(6)	0.011(1)	1.334(10)	0.602(1)	0.152(2)
*THRAP3*	0.386(2)	0.016(3)	1.245(5)	0.624(3)	0.170(3)
*YY1*	0.465(7)	0.019(6)	1.283(7)	0.654(6)	0.193(4)
*RHOA*	0.475(8)	0.013(2)	1.314(8)	0.611(2)	0.200(5)
*RNF-10*	0.441(5)	0.017(4)	1.334(11)	0.637(5)	0.236(6)
*QRICH1*	0.429(4)	0.020(7)	1.281(6)	0.659(7)	0.241(7)
*TARDBP*	0.394(3)	0.024(9)	1.183(3)	0.693(8)	0.269(8)
*SRSF3*	0.514(9)	0.022(8)	1.331(9)	0.727(9)	0.359(9)
*RPL13A*	0.615(12)	0.037(13)	1.087(2)	0.839(12)	0.445(10)
*TUBA1A*	0.552(10)	0.027(10)	1.424(13)	0.785(10)	0.513(11)
*DNAJC8*	0.711(15)	0.039(14)	0.992(1)	0.965(15)	0.583(12)
*GUSB*	0.646(13)	0.032(11)	1.344(12)	0.863(13)	0.593(13)
*ACTB*	0.583(11)	0.032(12)	1.529(14)	0.796(11)	0.608(14)
*GAPDH*	0.675(14)	0.046(15)	1.635(16)	0.882(14)	0.848(15)
*B2M*	0.748(16)	0.055(16)	1.576(15)	1.001(16)	0.977(16)

**Table 3:** Gene expression stability calculated by 5 algorithms in all BC cell strain samples

Gene	geNorm	NormFinder	BestKeeper	ΔCt method	ComprFinder
*THRAP3*	0.008(1)	0.354(1)	0.616(1)	0.300(1)	0.010(1)
*RHOA*	0.009(2)	0.447(5)	0.622(2)	0.426(7)	0.042(2)
*QRICH1*	0.013(3)	0.544(12)	0.664(3)	0.507(9)	0.111(3)
*SF1*	0.018(4)	0.509(8)	0.674(4)	0.777(13)	0.136(4)
*RNF10*	0.026(8)	0.507(7)	0.74(5)	0.501(8)	0.209(5)
*DNAJC8*	0.026(7)	0.377(2)	0.762(8)	0.419(6)	0.217(6)
*GUSB*	0.025(6)	0.523(10)	0.757(7)	0.402(5)	0.232(7)
*YY1*	0.027(9)	0.495(6)	0.773(9)	0.353(3)	0.254(8)
*RPL13A*	0.038(11)	0.393(3)	0.784(11)	0.639(11)	0.267(9)
*TARDBP*	0.019(5)	0.539(11)	0.744(6)	0.318(2)	0.268(10)
*GAPDH*	0.035(10)	0.573(13)	0.774(10)	0.606(10)	0.363(11)
*TRA2B*	0.039(12)	0.421(4)	0.918(12)	0.390(4)	0.386(12)
*SRSF3*	0.048(13)	0.510(9)	1.025(13)	0.716(12)	0.511(13)
*B2M*	0.058(14)	0.946(15)	1.084(14)	0.821(14)	0.767(14)
*TUBA1A*	0.067(15)	0.772(14)	1.374(16)	0.932(15)	0.879(15)
*ACTB*	0.077(16)	0.977(16)	1.175(15)	1.475(16)	0.901(16)

#### NormFinder analysis

Based on variance analysis to calculate the stable value of each gene, a higher NormFinder value indicates a less stably expressed gene. In the breast cancer tissue group, *TRA2B*, *RHOA*, and *THRAP3* were the most stable genes, and *DNAJC8*, *GAPDH,* and *B2M* were the most unstable genes (Table 2). In the breast cancer cell group, *THRAP3*, *DNAJC8,* and *RPL13A* were the three most stably expressed genes, while *TUBA1A*, *B2M*, and *ACTB* were the least stably expressed genes (Table 3). For all breast cancer tissue and cell line samples, *THRAP3*, *RHOA*, *QRICH1* were the most stably expressed genes, and *TUBA1A*, *B2M*, *ACTB* were the least stably expressed RGs (Additional file 5: Table S3).

#### BestKeeper analysis

To further analyze the expression stability of the RGs, BestKeeper was applied, in which a lower standard-value indicates a more stably expressed RG. As shown in Table 2, in the breast cancer tissue group *DNAJC8*, *RPL13A,* and *TARDBP* were the most stably expressed genes, while *ACTB*, *B2M,* and *GAPDH* were the least stably expressed genes (shown in Table 2). In the breast cancer cell line group, *THRAP3*, *RHOA*, and *QRICH1* were the three most stably expressed genes, while *B2M*, *ACTB,* and *TUBA1A* were the least stably expressed genes (shown in Table 3). For all samples combined, *DNAJC8*, *RPL13A*, and *TUBA1A* were the most stably expressed genes, while *GAPDH*, *RNF10,* and *ACTB* were the least stably expressed RGs (Additional file 5: Table S3).

#### ΔCt analysis

According to the ΔCt method, *TRA2B*, *RHOA,* and *THRAP3* were the most stably expressed genes, while *DNAJC8*, *GAPDH*, and *B2M* were the least stable genes in the breast cancer tissue group (Table 2), which was consistent with the analysis according to NormFinder. In addition, *THRAP3*, *TARDBP*, and *YY1* were the most stably expressed genes in the breast cancer cell lines, while *B2M*, *TUBA1A,* and *ACTB* were the least stably expressed genes (Table 3). For all samples combined, *THRAP3*, *RHOA*, and *QRICH1* were the most stably expressed genes, while *TUBA1A*, *B2M,* and *ACTB* were the least stable RGs (Additional file 5: Table S3).

#### A comprehensive ranking of the four methods examined

The ComprFinder algorithm was carried out to obtain a final score which was used to rank the potential RGs. In the breast tumor tissue group, the 3 most stably expressed RGs were *SF1*, *TRA2B,* and *THRAP3* (Table 2). In the breast cancer cell lines, *THRAP3*, *RHOA,* and *QRICH1* were the most stably expressed RGs (Table 3). For all samples combined, we ranked the RGs from the highest to the lowest stability as follows: *THRAP3* > *RHOA* > *QRICH1* > *YY1* > *TRA2B* > *RPL13A* > *SF1* > *SRSF3* > *GUSB* > *TARDBP* > *DNAJC8* > *RNF10* > *GAPDH* > *TUBA1A* > *B2M* > *ACTB*. Interestingly, the top 5 most stable genes (*THRAP3*, *RHOA*, *QRICH1*, *YY1*, and *TRA2B*) were novel RGs. In contrast, the traditionally used RGs *TUBA1A*, *B2M,* and *ACTB* were the least stably expressed RGs.

The research presented here confirmed that *THRAP3*, *RHOA*, *QRICH1*, *YY1*, and *TRA2B* were the most stable RGs in all samples with FS values of 0.064, 0.101, 0.122, 0.151, and 0.161, respectively (Additional file 5: Table S3). These promising results warranted further validation of the selected RGs.

### Validation of the selected genes (1): comparison of target gene profiles when using different normalized RGs

To verify the reliability of the selected RGs, the expression profiles of *MAPK3*, *MAPK9*, *FAAH*, and *HIF1A* were determined in different breast cancer tissues and cell lines. Our results indicated that *SF1*, *TRA2B*, and *THRAP3* were the top 3 stably expressed RGs in breast cancer tissues and that *THRAP3*, *RHOA*, and *QRICH1* were the top 3 stably expressed RGs in breast cancer cell lines. Moreover, five genes (*SF1*, *TRA2B*, *THRAP3*, *RHOA*, and *QRICH1*) were the top 5 stably expressed candidate RGs in all samples. Therefore, we considered the multi-RG combination *SF1* + *TRA2B* + *THRAP3* + *RHOA* + *QRICH1* as the most promising choice for breast cancer research (both in breast cancer tissues and cell lines). Thus, the multi-gene combinations including *SF1* + *TRA2B* + *THRAP3* + *RHOA* + *QRICH1*, *SF1* + *TRA2B* + *THRAP3*, *THRAP3* + *RHOA* + *QRICH1*, *SF1* + *THRAP3*, *THRAP3* + *RHOA*, and single RGs including *SF1*, *TRA2B*, *THRAP3*, *RHOA,* and *QRICH1* were compared. In addition, *ACTB*, *GAPDH*, and *ACTB* + *GAPDH* were also used for comparison with the novel RGs. In total, 13 different multi-RG combinations or single RGs were assessed. For multiple gene combinations, the geometric average of their Ct value was calculated. The relative gene expression level was calculated as 2^−ΔCt^, where ΔCt = Δ (Ct_Target gene_–Ct_RGs_).

**Fig. 3:**
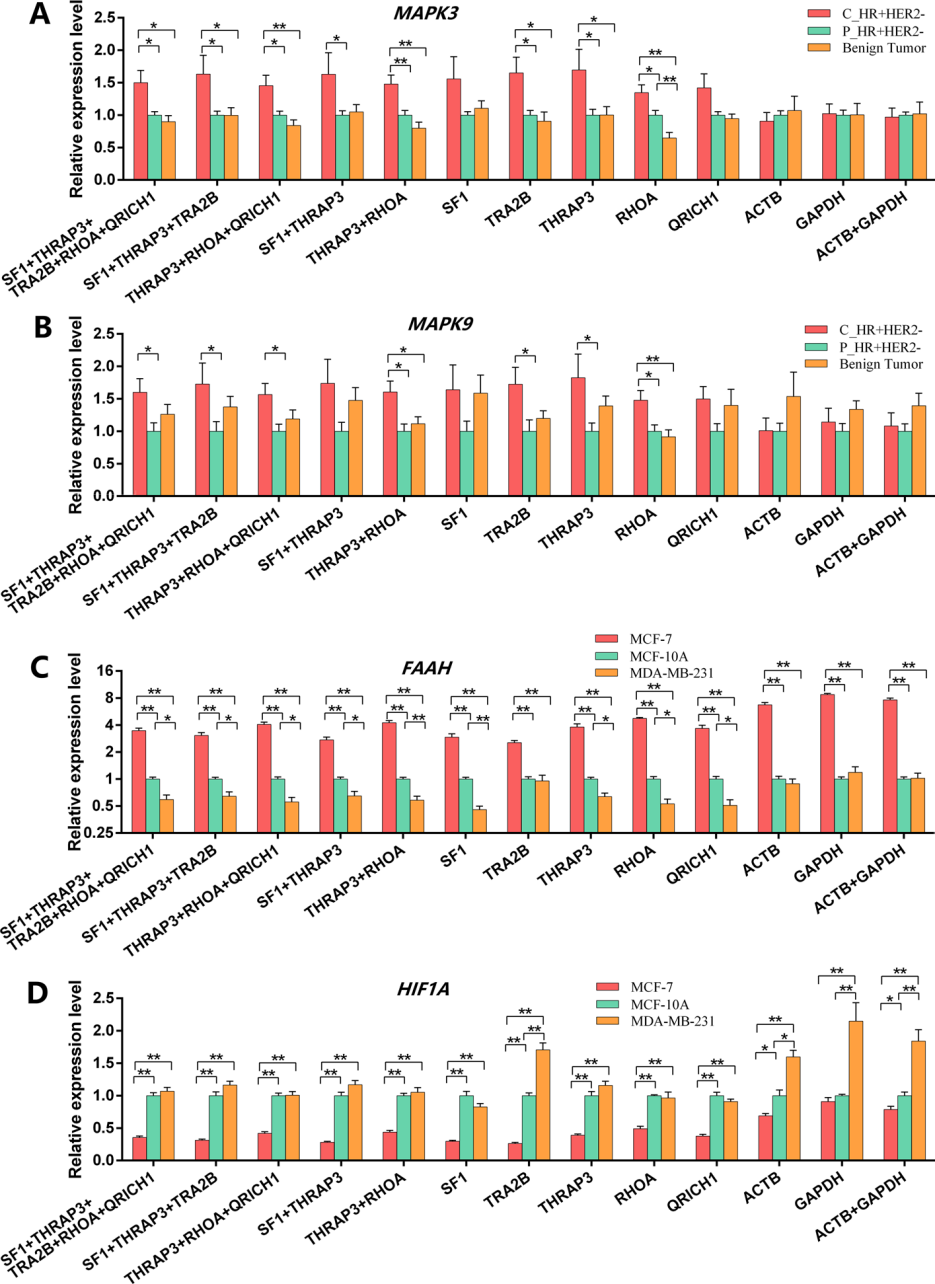
Relative expression levels normalized by 13 types of single- and multiple-RG combinations. Two target genes including MAPK3 (**A**) and MAPK9 (**B**) were determined in breast cancer (BC) tissue samples (C_HR + HER −, P_HR + HER −, and benign tumor), while FAAH (**C**) and HIF1A (**D**), were determined in BC cell lines (MCF-7, MCF-10A, and MDA-MD-231). Relative expression levels of each target gene were normalized by the most stable single RGs or multiple-RG combinations (SF1 + THRAP3 + TRA2B + RHOA + QRICH1, SF1 + THRAP3 + TRA2B, THRAP3 + RHOA + QRICH1, SF1 + THRAP3, TRA2B + RHOA, SF1, THRAP3, TRA2B, RHOA, and QRICH1) and the least stable single RGs or multiple-RG combinations (ACTB, GAPDH, and ACTB + GAPDH). The error bars represent the SEM, and the independent-sample *t*-test was performed between any two groups, *P < 0.05, **P < 0.01, *n* = 6 for each BC tissue group, n = 3 for each BC cell strain. The patterns of target gene expression were different between those normalized by the most stable RGs and those normalized by the least stable RGs

As shown in Fig. 3A, the expression of *MAPK3* was significantly higher (*P* < 0.05) in HR + HER2− cancer tissue than in para-carcinoma tissue or benign tumor tissue when assessed by 5 or 3 multi-gene RG combinations. However, the expression pattern of *MAPK3* changed when we used single or 2 multi-gene RG combinations, such as *SF1* + *THRAP3*, *SF1*, *RHOA*, or *QRICH1*. Importantly, when we investigated the least stably expressed RGs (*ACTB*, *GAPDH*, or *ACTB* + *GAPDH*), the expression of *MAPK3* was significantly changed compared with the most stably expressed RGs.

As shown in Fig. 3B, when using 3 or 5 multi-gene combinations, the expression level of the *MAPK9* gene was higher in HR + HER2 − cancer tissue than in para-carcinoma tissue (*P* < 0.05), while there was no significant difference between para-carcinoma tissue and benign tumor tissue. This may lead to small errors when using single or 2 multi-gene combinations. For example, when the less stably expressed genes *ACTB*, *GAPDH*, or *ACTB* + *GAPDH*, were used for data normalization, the expression of *MAPK9* did not show a clear expression trend compared with those of 3 or 5 multi-gene combinations.

In breast cancer cell lines, when the optimal RG combinations *SF1* + *TRA2B* + *THRAP3* + *RHOA* + *QRICH1*, *SF1* + *TRA2B* + *THRAP3*, or *THRAP3* + *RHOA* + *QRICH1* were used for normalization, the expression of *FAAH* was highest in MCF-7 cells, followed by MCF-10A cells, and was least in MDA-MB-231 cells (Fig. 3C). When *ACTB*, *GAPDH*, or *ACTB* + *GAPDH* were used for normalization, the expression of *FAAH* was not significantly different between MCF-10A and MDA-MB-231 cells.

The expression of *HIF1A* in breast cancer cells was higher (*P* < 0.01) in MCF-10A and MDA-MB-231 cells than in MCF-7 cells, while no significant difference was found between MCF-10A and MDA-MB-231 cells when using the 3 or 5 RG combinations (*SF1* + *TRA2B* + *THRAP3* + *RHOA* + *QRICH1*, *SF1* + *TRA2B* + *THRAP3,* or *THRAP3* + *RHOA* + *QRICH1*) for normalization (Fig. 3D). However, when *ACTB* or *GAPDH* (the less stably expressed RGs) were used, we found that *HIF1A* expression was significantly higher in MDA-MB-231 than in MCF-7 or MCF-10A cells.

The complete relative expression levels (2^−ΔCt^) of *MAPK3*, *MAPK9*, *FAAH*, and *HIF1A* genes normalized using all 13 types of single or multiple-RG combinations are listed in Additional file 6: Table S4 and Additional file 7: Table S5.

### Validation of the selected genes (2): the relationship among different normalized RGs

Based on the method described in our previous study [28], the relationship among different normalized RGs was explored. As shown in Additional file 2: Fig. S2, there was a high correlation (*R*^2^ from 0.815 to 0.979 in breast cancer tissues, and *R*^2^ from 0.927 to 0.995 in breast cancer cell lines) between stable RGs and *SF1* + *TRA2B* + *THRAP3* + *RHOA* + *QRICH1*. There was also a moderate-to-high correlation (R^2^ from 0.621 to 0.709 in breast cancer tissues, and R^2^ from 0.600 to 0.916 in breast cancer cell lines) between unstable RGs and *SF1* + *TRA2B* + *THRAP3* + *RHOA* + *QRICH1*. There were few differences between the most stably expressed RGs and the least stably expressed RGs. Therefore, we performed additional analyses of their normalized efficacy, including a correlation analysis on the *p*-value yielded by the *t*-test analysis (see Method section).

**Fig. 4:**
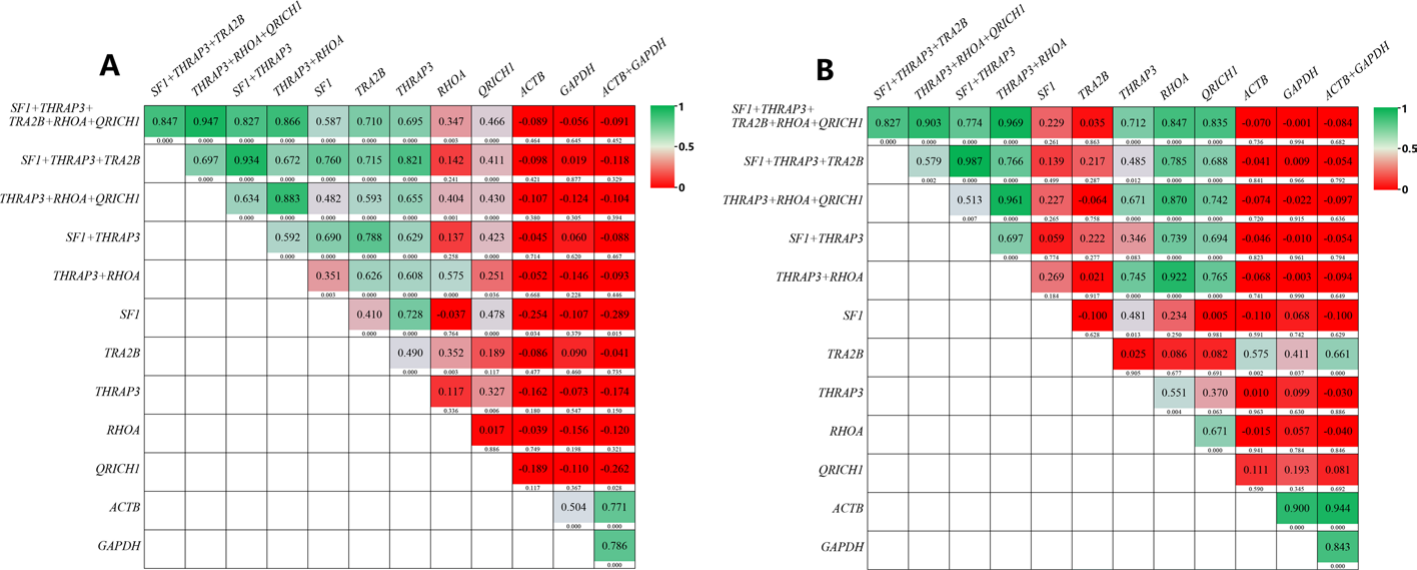
Heat map of correlation coefficients of detection efficiency when using different normalized RGs. Detection efficiency represents the quality of normalized results (the *P*-value of independent-samples *t*-test). Two target genes, *MAPK3* and *MAPK9*, were detected in all 66 breast cancer (BC) tissue samples (**A**) and two target genes *FAAH* and *HIF1A* were detected in 21 BC cell line samples (**B**) and were normalized by different types of RGs. The number in each color block is the correlation coefficient (R-value), and the number below each color block is the *P*-value of the corresponding R-value

As shown in Fig. 4A, in breast cancer tissues, the normalized results using *SF1* + *TRA2* + *THRAP3* (*R*^2^ = 0.847, *P* < 0.001), *THRAP3* + *RHOA* + *QRICH1* (*R*^2^ = 0.947, *P* < 0.001), *SF1* + *THRAP3* (R^2^ = 0.827, *P* < 0.001), or *THRAP3* + *RHOA* (*R*^2^ = 0.866, *P* < 0.001) displayed a high correlation with *SF1* + *TRA2B* + *THRAP3* + *RHOA* + *QRICH1* suggesting that they had extremely similar normalization capabilities. *SF1*, *TRA2B,* and *THRAP3* displayed a moderate correlation (*R*^2^ > 0.5), while *RHOA* or *QRICH1* displayed a weak correlation (*R*^2^ < 0.5) with *SF1* + *TRA2B* + *THRAP3* + *RHOA* + *QRICH1*. There was a poor correlation between less stably expressed RGs (*ACTB*, *GAPDH,* or *ACTB* + *GAPDH*) and *SF1* + *TRA2B* + *THRAP3* + *RHOA* + *QRICH1*. Similar results were found for the breast cancer cell lines (Fig. 4B**)**. The complete *p*-value results yielded by *t*-test analysis are given in Additional file 8: Table S6 and Additional file 9: Table S7.

## Discussion

### The importance of reference genes

The qRT-PCR technique is one of the most valuable and reliable research tools to quantify the expression of a target gene under different experimental conditions. Proper use of RGs is necessary to get a reliable estimate of gene expression in different types of breast cancer tissues and cell lines to avoid detecting variations that are not cancer-specific [29–31]. Therefore, the selection of the appropriate RGs for breast cancer research is important when using qRT-PCR to quantify gene expression. Many studies use a single endogenous control for normalization, which can influence the statistical results and may lead to erroneous data interpretation [2, 32]. In fact, in the present study, no single RGs were identified that were stably expressed in all tissues or cell types across different types of breast cancer [7, 33, 34].

Theoretically, RGs should be stably expressed in all samples, and their expression levels should be unaffected by the external environment, e.g., during tumorigenesis [35]. The selection and validation of RGs have to be corroborated by using a large number of samples [36, 37]. To implement this idea, in this study we collected a large number (*n* = 87) of samples including 6 types of breast cancer tissues and 7 types of breast cancer cell lines. This allowed us to obtain strong results and conclusions. There was a great diversity of samples in our study for the following reasons: (a) both benign and malignant tumor types were chosen; (b) breast cancer samples following neoadjuvant chemotherapy were included; (c) the breast cancer cell lines included overexpression and knock-down groups. With the above caveats explained, we propose that we have identified combinations of RGs that have high applicability in breast cancer research and treatment.

### Confirm the application of RGs

In our study, we used five algorithms to determine the stability of the expression of 16 candidate RGs across several different types of breast tumors and breast cancer cell lines. We found, that *SF1* was the most stably expressed gene according to geNorm and ComprFinder but ranked fifth by NormFinder and fourth by BestKeeper and the ΔCt method in breast cancer tissue samples. On the other hand, NormFinder and the ΔCt method recommended *TRA2B* as most appropriate for normalizing expression in breast cancer tissue samples. Surprisingly, *THRAP3* was the most stably expressed gene according to all five algorithms in breast cancer cell lines.

The ideal reference gene shows a constant level of expression that does not vary by tissue or cell type and is not influenced by the treatment that is applied. However, numerous studies have shown that no gene is permanently and stably expressed under all circumstances. Therefore, reference genes must be evaluated for each breast cancer type and each experimental setup and multiple gene combinations must be used. Even for the same algorithm, the results varied between breast cancer tissues and cell lines. The top three genes for breast cancer tissues and cell lines were *SF1* + *TRA2B* + *THRAP3* and *THRAP3* + *RHOA* + *QRICH1*, respectively, and therefore a total of 5 RGs (*SF1*, *TRA2B*, *THRAP3*, *RHOA*, *QRICH1*) should be considered. Unfortunately, determination of the expression of all five RGs simultaneously would require a lot of effort.

There are no specific literature reports prescribing how many candidate RGs should be used for qRT-PCR-dependent studies [38]. In particular, it is unknown which single or multiple gene combinations (*SF1* + *TRA2B* + *THRAP3* + *RHOA* + *QRICH1*, *SF1* + *TRA2B* + *THRAP3*, *THRAP3* + *RHOA* + *QRICH1*, *SF1* + *THRAP3*, *THRAP3* + *RHOA*, *SF1*, *TRA2B*, *THRAP3*, *RHOA*, or *QRICH*) should be used. Considering that our results indicate that the single gene performances of both novel and traditional RGs are not adequate, we propose that these types of studies should not be based on the use of single RGs, even if they are top-level RGs. The double gene combinations *SF1* + *THRAP3* and *THRAP3* + *RHOA* showed similar gene expression profiles consistent with *SF1* + *TRA2B* + *THRAP3* + *RHOA* + *QRICH1*, *SF1* + *TRA2B* + *THRAP3,* and *THRAP3* + *RHOA* + *QRICH1*. However, the *SF1* + *THRAP3* combination behaved similarly to the 3 or 5-gene combinations except for the *MAPK3* and *MAPK9* expression. Meanwhile, the *THRAP3* + *RHOA* combination behaved similarly to the 3 or 5-gene combinations except for the *MAPK9* expression. Therefore, considering the need for normalization accuracy, double RGs are not the optimal choice either.

The expression pattern of target genes was the same when 3-gene combinations or 5-gene combinations were used and they can be applied to various factors in breast cancer research. However, 3 RGs is a more manageable number for normalizing qRT-PCR experiments than 5 RGs. Therefore, we recommend that *SF1* + *TRA2B* + *THRAP3* and *THRAP3* + *RHOA* + *QRICH1* be adopted as the RG combinations for breast cancer tissue and cell line research, respectively. In the case of studies including both breast cancer tissue and cell line research, the *THRAP3* + *RHOA* + *QRICH1* combination would be optimal.

### The previous RGs comparison

The target genes that were used in this study are involved in different biological processes of breast carcinogenesis and metastasis. Particularly, tumorigenesis, proliferation, apoptosis, and invasion are associated with many genes and signaling pathways. For example, genes such as *MAPK3* and *MAPK9* encoding MAP kinases of the ERK signal pathway participate in transcription factor regulation of many biological processes [39, 40]. Recently, novel results have indicated proteins that serve important roles during the process of cancer development. FAAH is a membrane-bound protein belonging to the serine hydrolase family of enzymes that plays a significant role in the termination of signaling of fatty acid amides (FAAs), a class of bioactive lipids, both in the central nervous system and in some cancer tissues [41]. Hypoxia-inducible factors (such as HIF1A) play an important role in the development of tumors, thus the study of these factors is indispensable for cancer research [42, 43]. Therefore, to confirm the roles of these genes on the vital regulatory mechanisms in breast cancer, we compared the potential role of novel RGs (*SF1*, *TRA2B*, *THRAP3*, *RHOA*, and *QRICH1*) vs. traditional RGs (*ACTB*, and *GAPDH*) in the normalization of target gene expression.

### Our proposal

In this study, we did not merely verify the use of conventional RGs, but also identified and selected more appropriate novel RGs for breast cancer research. Our results show, that the use of a single RGs should be avoided for breast cancer research. Similarly, the use of double RGs is not recommended. These findings are similar to what has been suggested in most of the studies using transcriptomic datasets [44]. As far as we know, only one previous study reported on the role of RGs in the normalization of breast cancer gene expression studies. The previous studies of RGs used traditional RGs, and other breast cancer studies were also based on traditional RG [3, 7]. In the present study, a large number of biological samples were provided for determination and validation, and multiple algorithms were used for evaluation, with the RNA-seq dataset being used for prediction and selection. Therefore, in terms of both the number and quality of RGs, this study is a significant step forward from previous studies. Our results suggest that the recommended number of RG is at least three for breast cancer tissues or cell lines. Nevertheless, these promising results require further verification of target genes in order to obtain more reliable data sets.

### Limitations and future research suggestions

Although this study was based on a large amount of transcriptome data to predict the new RGs, and a large number of breast cancer samples were used for confirmation and verification, we still cannot guarantee that our research results apply to all breast cancer types, especially those rare disease types, such as medullary breast carcinoma. In addition, mutations of gene expression always exist, and the number of samples in our study was limited. Therefore, our final recommendation may not be an absolute perfect choice, but a relatively better choice.

Sequencing technology is widespread with the development of genomics, and large amounts of accumulated data need to be interpreted from a multi-disciplinary perspective in order to choose suitable RGs [45]. Some emerging technologies and methods for data mining require us to borrow and learn, such as the multi-objective Particle Swarm Optimization [46], and the meta-heuristic optimization algorithm [47]. In future work, more novel algorithms need to be developed for explaining how normalization affects breast cancer expression data gathered by qRT-PCR, which will allow us to improve the accuracy and standardization across study systems.

## Conclusions

In this study, we tested 16 different candidate RGs in six different breast cancer tissues and seven breast cancer cell lines, using five different statistical algorithms for evaluation. Our results indicate that *SF1* + *TRA2B* + *THRAP3* and *THRAP3* + *RHOA* + *QRICH1* are promising RG combinations for efficient gene normalization under different conditions. Furthermore, the availability of these RGs and the stability of their expression in various tumor tissues and cells will allow performing future studies focusing on genes essential for breast cancer biology, and choosing a reliable and appropriate RG combination will allow more accurate assessments of differential gene expressions in breast cancer research.

## Methods

### Breast cancer tumor

Breast tumor and para-carcinoma tissues were supplied by the Breast Tumor Biobank of the Three Gorges Hospital Affiliated with Chongqing University. Fresh tissues were obtained from patients with written informed consent and with permission of the Three Gorges Hospital Affiliated with Chongqing University Clinical and the Laboratory Research Ethical Council. All tissues were stored frozen at – 80 ℃ after pathologic evaluation. We collected a total of 66 tissue samples including benign tumor tissues (*n* = 6), as well as tissues from four subtypes of breast cancer including HR + /HER2 − (*n* = 6), HR + /HER2 + (*n* = 6), HR −/HER2 − (*n* = 6), HR −/HER2 + (*n* = 6), and their paired para-carcinoma tissues (*n* = 6 each) from 24 patients who were diagnosed with breast cancer and from 6 patients who were diagnosed with breast cancer and then were treated with NAC before surgery. The para-carcinoma tissue samples had been taken from outside of the histopathological tumor border (3 cm) in the same excisional biopsy specimen. The clinical patient information is shown in Additional file 10: Table S8.

### Cell lines and related treatment

Breast cancer cell lines T-47D, MDA-MB-231, and MDA-MB-486 were purchased from the Cell Bank of the Type Culture Collection of the Chinese Academy of Sciences (Shanghai, China). MCF-10A and MCF-7 cell lines were purchased from the American Type Culture Collection (ATCC, Manassas, USA). MDA-MB-231 and MDA-MB-486 cells were cultured in Leibovitz's L-15 Medium (L-15, Gibco, USA). T-47D cells were cultured in Dulbecco's modified Eagle medium, containing high glucose and pyruvate without glutamine (DMEM, Gibco, USA). MCF-10A cells were cultured in DMEM: Nutrient Mixture F-12 (DMEM/F-12, Gibco, USA) and MCF-7 cells were cultured in Minimum Essential Medium supplemented with 0.01 mg/ml bovine insulin (MEM, Gibco, USA). Moreover, we have constructed the MDA-MB-231 cell lines overexpressing CNR2 or CNR2 knock-down using lentiviruses (Genechem, Shanghai, China). All culture media were supplemented with 20U/mL penicillin, 100 mg/mL streptomycin, and 10% heat-inactivated fetal bovine serum (FBS, Gibco, Australia). Cells were grown at 37 ℃ in a humidified atmosphere including 5% CO_2_. At the end-point of each experiment, the final pH of the supernatant was always measured by a digital pH-meter (pH 301, HANNA Instruments, USA).

### Total RNA extraction and cDNA synthesis

Total RNA was isolated with RNAiso Plus (Takara, Dalian, China) using the phenol–chloroform method. Extracted RNA was quantified using Nanodrop One (ThermoFisher, Wilmington, USA) and its integrity was checked on a 1% agarose gel. Only RNA samples with A260/A280 ratios between 1.9 and 2.2 and A260/A230 ratios greater than 2.0 were used for cDNA synthesis. Total RNA (1 μg) was reverse-transcribed into cDNA using random primers or an oligo dT primer using a PrimeScript RT reagent Kit with gDNA Eraser (Takara, Dalian, China), according to the manufacturer’s protocol [48]. All cDNA samples were diluted 1:8 with RNase-free water and stored at – 20 ℃.

### Selection of candidate reference genes

**Fig. 5:**
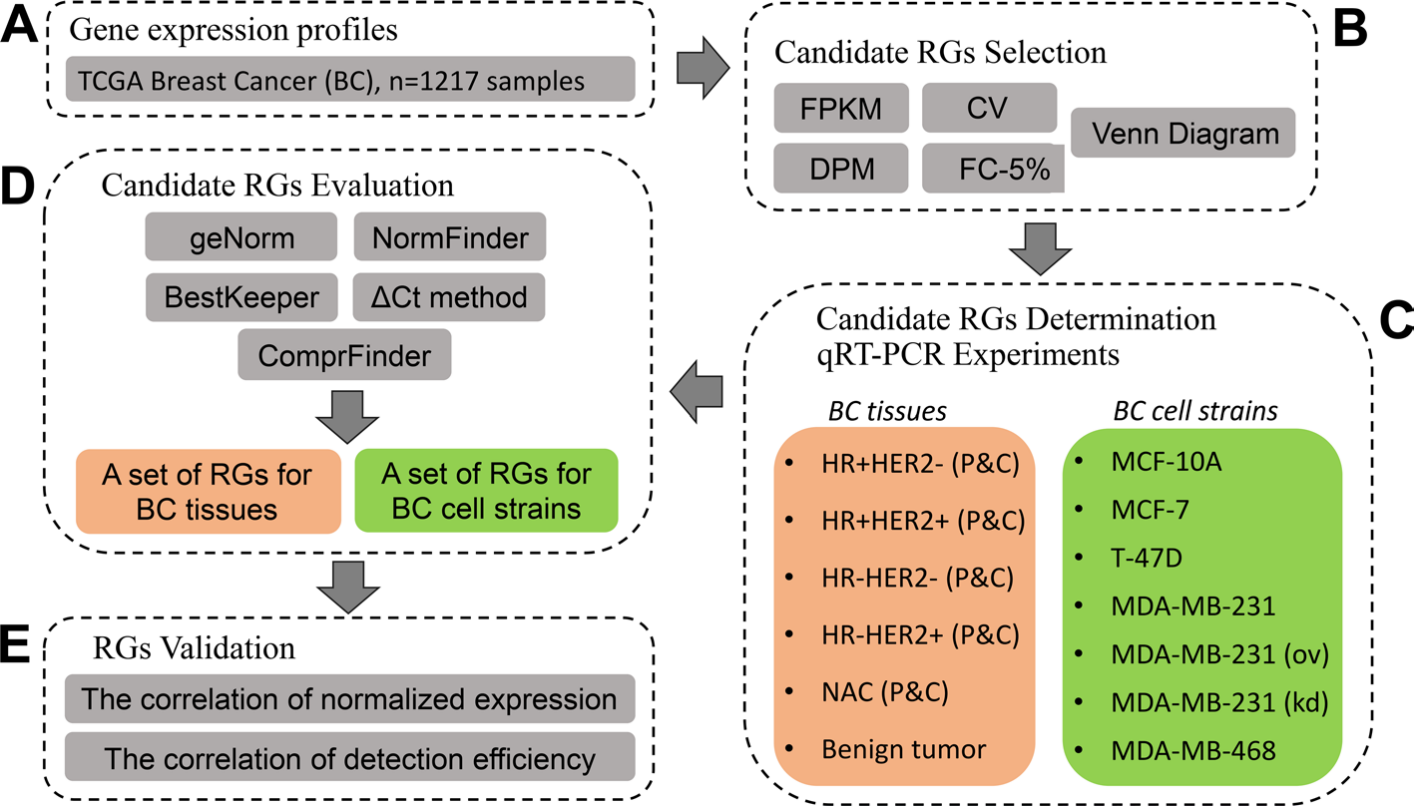
The workflow of this study. (**A)** The gene expression profiles of 1217 breast cancer (BC) samples were obtained from the TCGA public database; (**B)** Four indexes, including FPKM, CV, DPM, and FC-5%, were used to select candidate RGs and Venn diagram analysis identified the RGs common to these indexes; (**C)** qRT-PCR experiments were carried out on various BC tissue specimens (n = 66) and BC cell lines (n = 21). P, adjacent tissues; C, cancer tissues. Six biological replicates in each tissue group and three replicates for each BC cell line were used in this study. ov, overexpression; kd, knock-down. (**D**) Candidate RGs were identified and evaluated by 5 public algorithms including geNorm, NormFinder, BestKeeper, ΔCt method, and ComprFinder. (**E**) The selected RGs were validated using 4 target genes (MAPK3, MAPK9, FAAH, and HIF1A). After being normalized by different RGs, the patterns of target gene expression were compared. The capabilities of different types of RGs were tested by correlation analysis

The transcriptome sequencing dataset of 1217 breast cancer samples was downloaded from the TCGA database (https://www.cureline.com/the-cancer-genome-atlas.html) (Fig. 5A). After obtaining the gene fragments per kilobase of exon model per million mapped reads (FPKM), transcripts that exhibited low levels (FPKM = 0 appearing over 61 times in 1217 transcriptome profiles, 1217 × 5% = 60.85) were removed. According to the FPKM value of every gene in all transcriptome profiles [49], the coefficient of variation (CV) [50], dispersion measure (DPM, calculated using a jar package from Pan et.al [51], and FC-5% were calculated to screen for novel RGs (shown in Fig. 5B). The CV was defined as the CV value of the 1217 FPKM values of every gene. The DPM parameter was introduced for the identification of the RGs on the Pattern Gene Finder [51]. The FC-5% was defined as the fold change between the top 5% high expression levels divided by the bottom 5% within 1217 profiles. The standard criteria of candidate RGs were relatively high expression levels and low variation according to the results from FPKM, CV, DPM, and FC-5% analyses. Briefly, the evaluation parameter criteria FPKM ≥ 10, CV ≤ 40%, FC-5% ≤ 5, and DPM ≤ 0.3 were set for seeking novel candidate RGs. In addition to the software mentioned above, MS Excel 2019 was used for these analyses.

Furthermore, two frequently used RGs (*ACTB* and *GAPDH*) and four RGs (*GUSB*, *RPL13A*, *TUBA1A*, and *B2M*) from previous studies were also assessed along with the novel candidate RGs. All RGs were amplified using qRT-PCR for subsequent determination and validation. The probability density curves were drawn using Matlab scripts from our previous study [28]. Venn diagram analysis was performed using a webtool (http://www.omicshare.com/tools).

### Primer design and amplification efficiency analysis

The sequences of all genes used in this study were obtained from the National Center for Biotechnology Information (NCBI, https://www.ncbi.nlm.nih.gov/). Using Primer-BLAST, primers were designed for all transcripts, with T_m_ values around 60 °C, GC percent 45–55%, primer lengths of 18–24 bp, and product length of 80–250 bp. Primers were analyzed with Oligo Analyzer v3.1 (https://eu.idtdna.com/calc/analyzer) to detect potential self-annealing and formation of heterodimers [52]. The primers were synthesized by the Beijing Genomics Institute (Beijing, China). Primer specificities were confirmed by melting curve analysis.

### qRT-PCR analysis

All qRT-PCR runs were carried out in a qTower2.2 PCR System (Analytik Jena, Germany). Reaction mixtures containing 7.5 μL TB Green Premix Ex Taq II (2 ×, Tli RNaseH Plus), 0.3 μL ROX Reference Dye II (50 ×, TaKaRa, Dalian, China), 1.5 μL cDNA, 0.6 μL each of forward and reverse primers (final concentration 1 μM), and 4.6 μl nuclease-free water were prepared in MicroAmp fast optical 96-well plates (ThermoFisher, USA). Amplification conditions were set as follows: 95 ℃ for 30 s, followed by 40 cycles of 95 ℃ for 5 s and 60 ℃ for 34 s. Melting curve analysis was performed from 60 to 95 ℃. Reaction mixtures containing no template were used as negative controls. All samples were analyzed with three technical replicates. To test the amplification efficiency of each paired primer, serial tenfold dilutions (1:10^3^–1:10^10^) of the primer corresponding to PCR product were used to generate a standard curve [53]. The coefficient of determination (*R*^2^) and slope (S) values were calculated from the standard curves and primer efficiencies (E) were calculated as ^10^(1/S)−1. The qRT-PCR experiments and analyses in this study were performed according to the Minimum Information for Publication of Quantitative Digital PCR Experiments (MIQE) guidelines [54].

### Analysis of gene expression stability

The cycle threshold (Ct) results from all runs were integrated into a data matrix. Then the data matrix was evaluated by four algorithms: geNorm, NormFinder, ΔCt method, and BestKeeper. Finally, the gene stability values from the above four algorithms were further evaluated by the ComprFinder method (shown in Fig. 5D).

### Validation of the candidate reference genes

To verify the reliability of the stable RGs, four target genes including *MAPK9* and *MAPK3* from the extracellular signal-regulated kinase (ERK) signal pathway, and two other vital functional genes (*FAAH,* encoding fatty acid amide hydrolase, and *HIF1A,* encoding hypoxia-inducible factor 1-alpha) were chosen for validation (shown in Fig. 5E). These target genes play an important role in the initiation and metastasis of breast cancer [42, 55–58]. The independent-sample *t*-test was performed using Microsoft Excel, and the graphs were plotted using GraphPad Prism 7. The results are presented as mean ± standard error of the mean (SEM), *P < 0.05, **P < 0.01. For multiple gene combinations, the geometric mean of their Ct values was calculated. The relative expression levels were calculated using the 2^−ΔΔCt^ method. To further evaluate the internal relationship of these different types of single- or multi-RG combinations, correlation analysis was performed as previously described [28]. Additionally, correlation analysis was also performed on the *p*-value dataset yielded in *t*-test analysis under different types of normalized factors.

## Supplementary Information

**Additional file 1:** **Figure S1.** Melting curves for the 12 candidate RGs and 3 target genes

**Additional file 2:** **Figure S2.** Heat map of correlation coefficients of relative expression levels based on different normalized RGs.

**Additional file 3:** **Table S1.** The clinical information of all samples in this study.

**Additional file 4:** **Table S2.** Primer sequences and amplicon information of candidate RGs and target genes for qRT-PCR.

**Additional file 5:** **Table S3.** Ct values of the 16 candidate RGs in all samples.

**Additional file 6:** **Table S4.** Gene expression stability calculated by 5 algorithms in all BC tissue and cell line samples.

**Additional file 7:** **Table S5.** Relative expression levels of MAPK3 and MAPK9 genes normalized by 13 types of single or multiple gene combinations of RGs in 66 BC tissue samples.

**Additional file 8:** **Table S6.** Relative expression levels of FAAH and HIF1A genes normalized by 13 types of single or multiple gene combinations of RGs in 21 BC cell strain samples.

**Additional file 9:** **Table S7.** *P*-value of *t*-test of target gene expression levels between different 11 levels of BC tissue samples.

**Additional file 10:** **Table S8.** *P*-value of *t*-test of target gene expression levels between different 7 levels of BC cell strain samples.

## Data Availability

The datasets used and/or analyzed during the current studies are available from the corresponding author on reasonable request.

## Abbreviations

RG: Reference gene
HR: Hormone receptors
HER-2: The human epidermal growth factor receptor 2
NAC: Neoadjuvant chemotherapy
ACTB: Beta-actin
GAPDH: Glyceraldehyde-3-phosphate dehydrogenase
GUSB: Beta-glucuronidase
RPL13A: Ribosomal protein L13a
TUBA1A: Tubulin alpha 1a
TCGA: The Cancer Genome Atlas
SF1: Splicing factor 1
TARDBP: TAR DNA binding protein
THRAP3: Thyroid hormone receptor associated protein 3
QRICH1: Glutamine rich 1
TRA2B: Transformer 2 beta homolog
SRSF3: Serine and arginine rich splicing factor 3
YY1: YY1 transcription factor
DNAJC8: DnaJ heat shock protein family (Hsp40) member C8
RNF10: Ring finger protein 10
RHOA: Ras homolog family member A
FPKM: Fragments per kilobase of exon model per million mapped reads
CV: Coefficient of variation
DPM: Dispersion measure
NCBI: The National Center for Biotechnology Information
MIQE: The Minimum Information for Publication of Quantitative Digital PCR Experiments
Ct: The cycle threshold
MAPK9: Mitogen-activated protein kinase 9
MAPK3: Mitogen-activated protein kinase 3
HIF1A: Hypoxia-inducible factor 1 subunit alpha
FAAH: Fatty acid amide hydrolase
