# Supplementary material for: Public transcriptome database-based selection and validation of reliable reference genes for breast cancer research
Source: Biomed Eng Online. 2021 Dec 11;20:124. doi: 10.1186/s12938-021-00963-8 (PMC8665499; doi:10.1186/s12938-021-00963-8)
Supplement: Supplementary file 1 — Additional file 1: Figure S1. Melting curves for the 12 candidate RGs and 3 target genes [file 12938_2021_963_MOESM1_ESM.pdf]

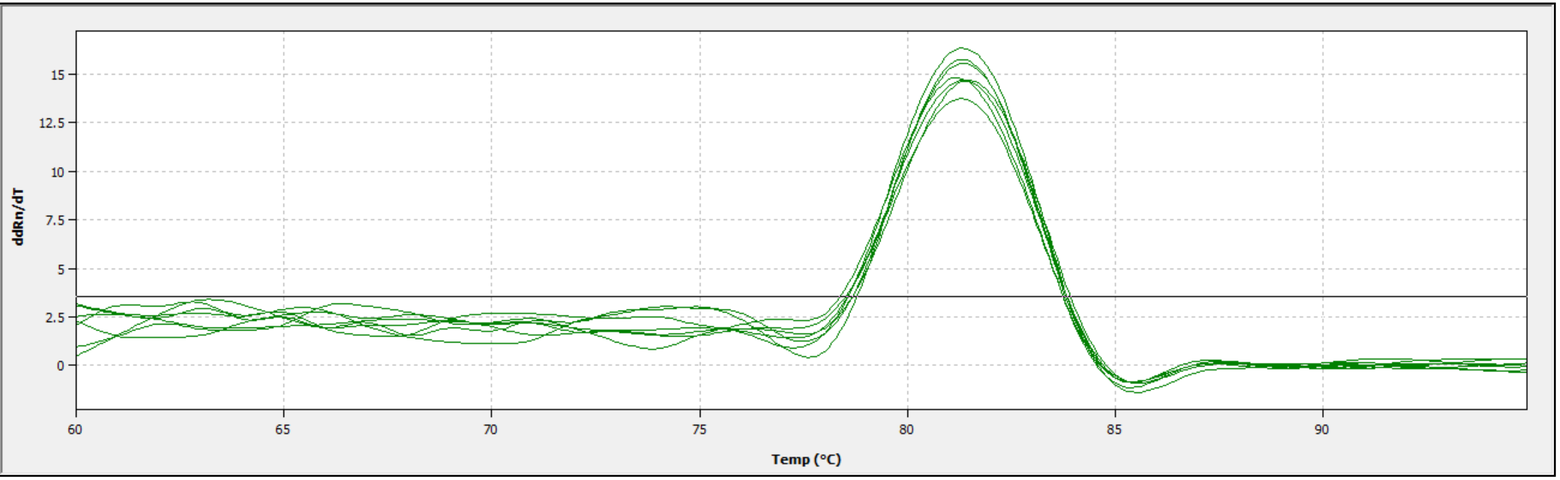

DNAJC8

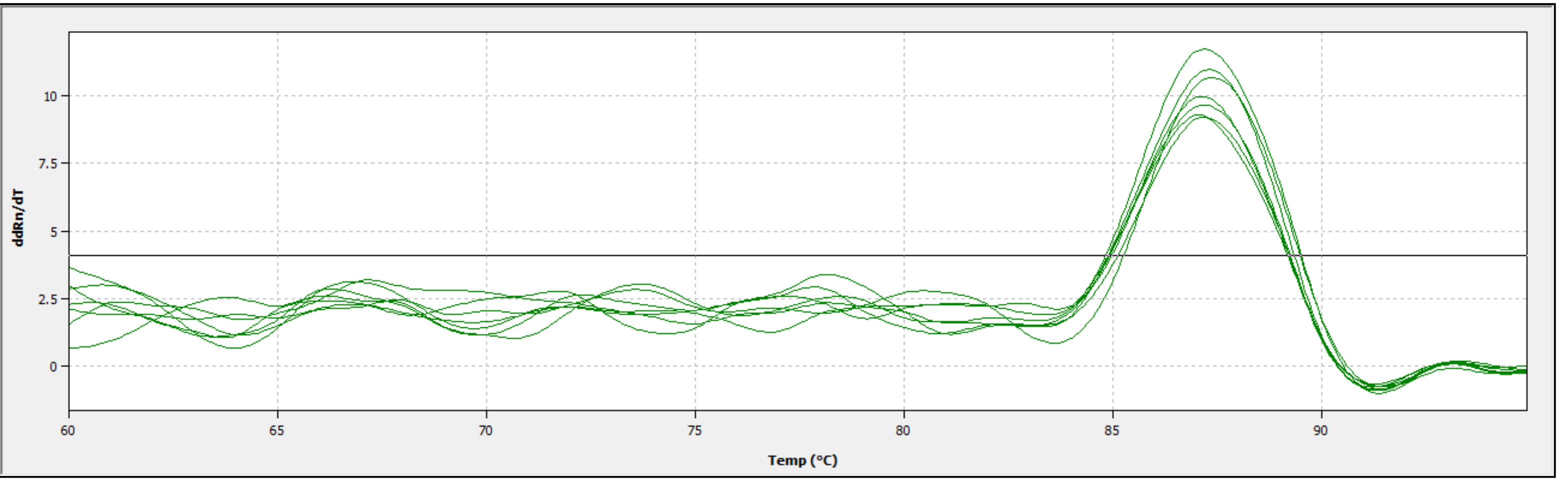

ACTB

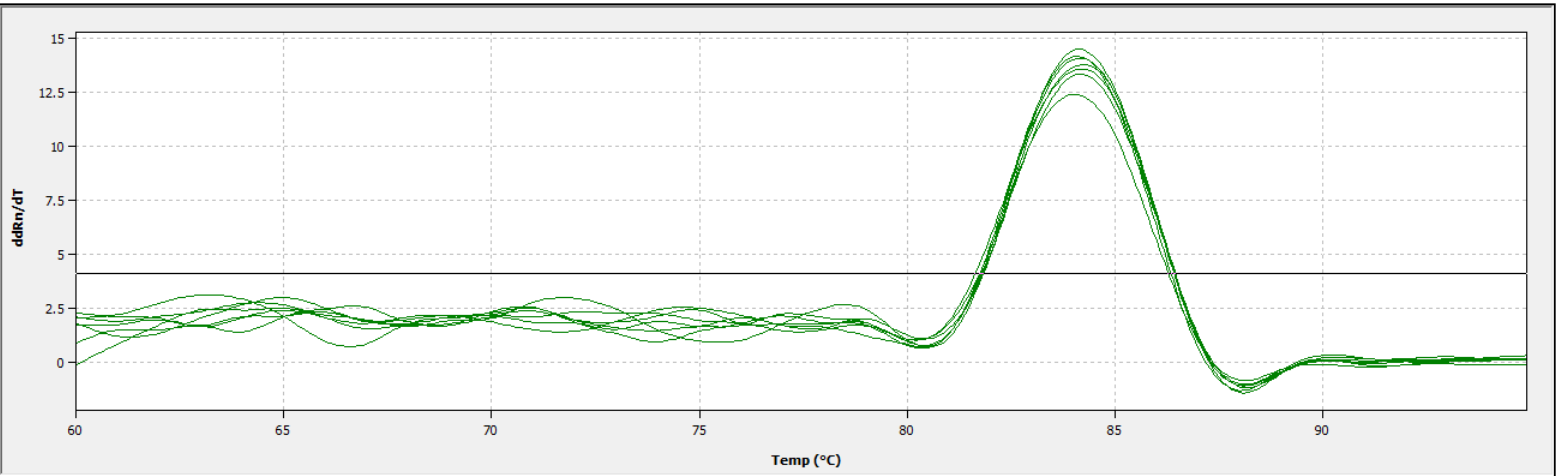

GAPDH

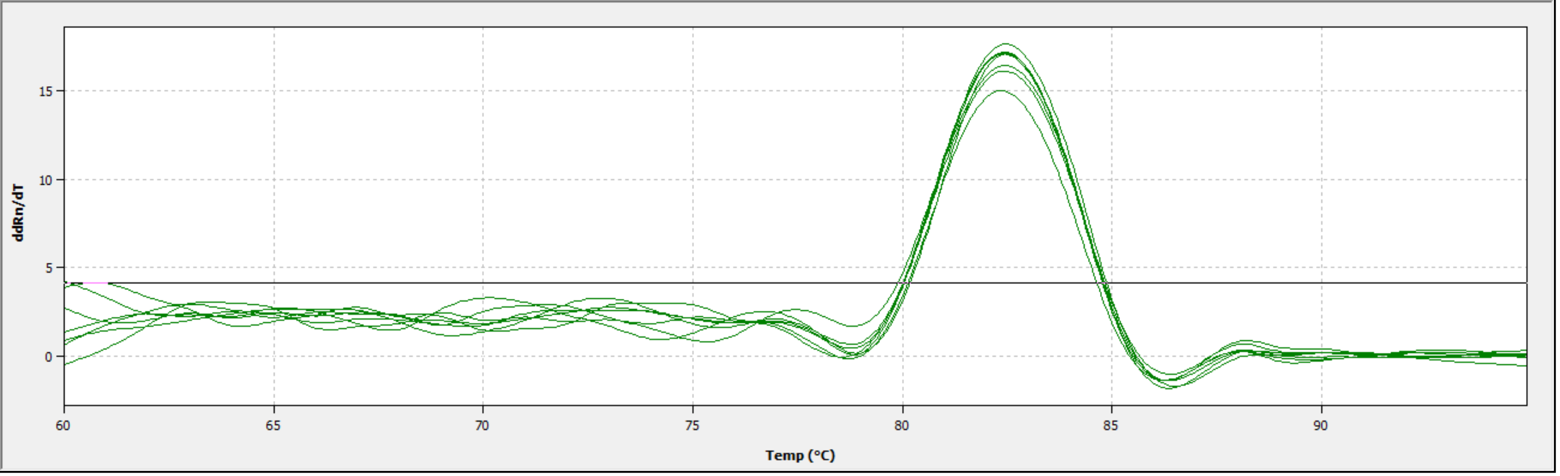

GUSB

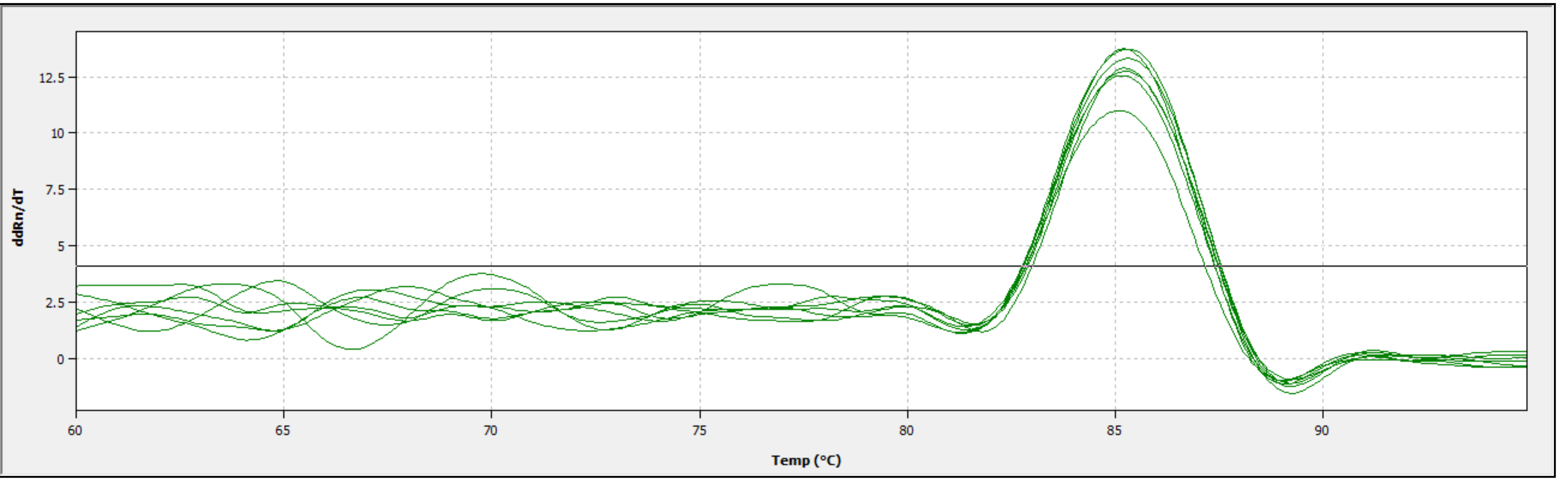

RPL13A

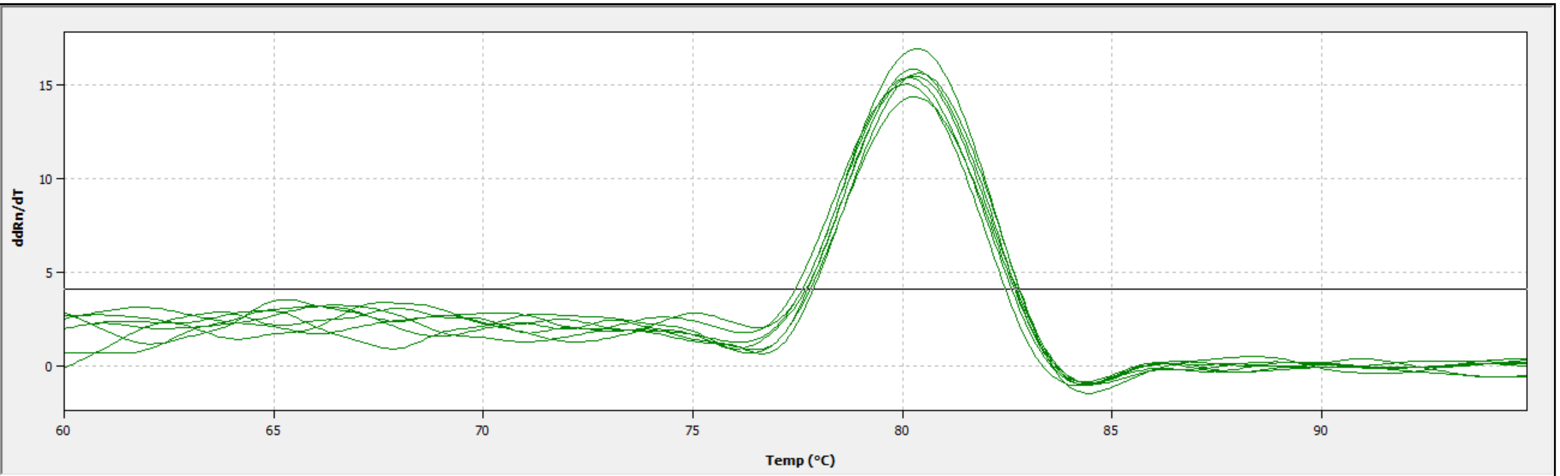

TUBA1A

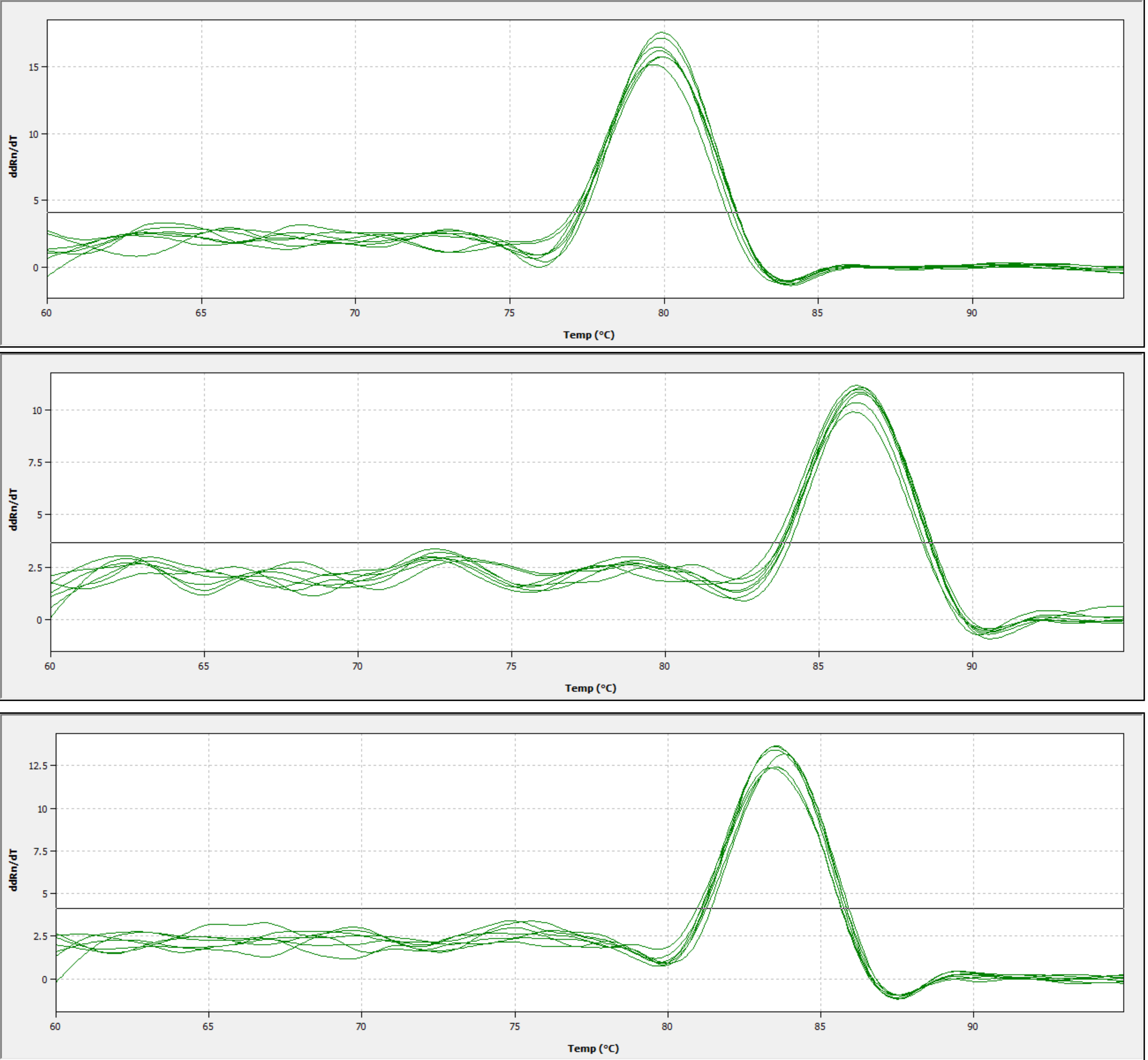

B2M

SF1

TARDBP

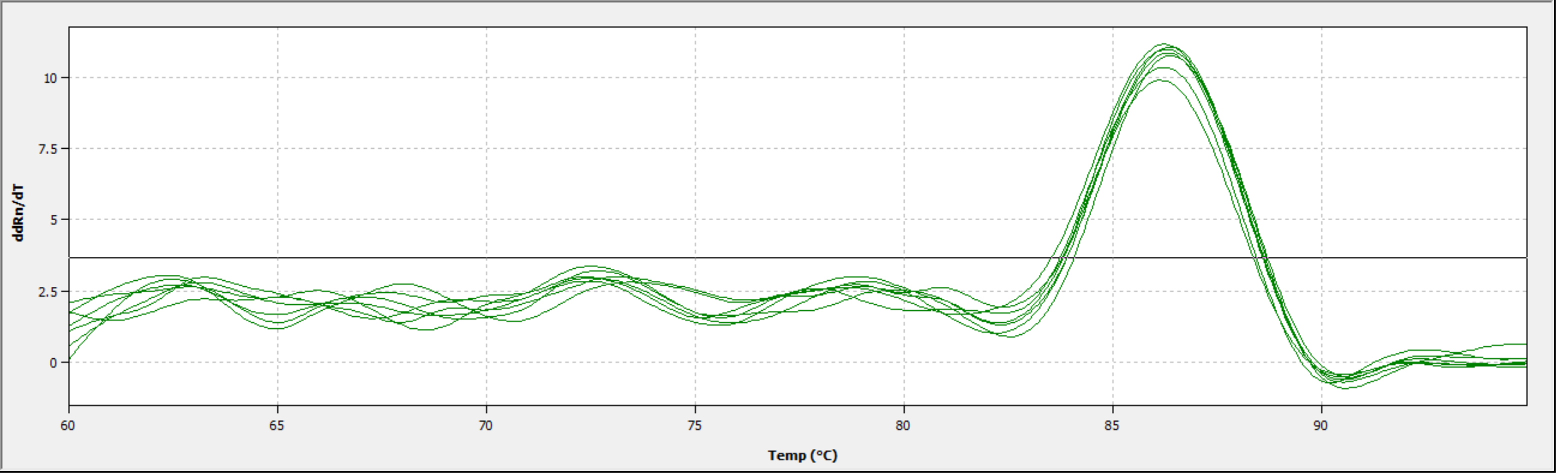

YY1

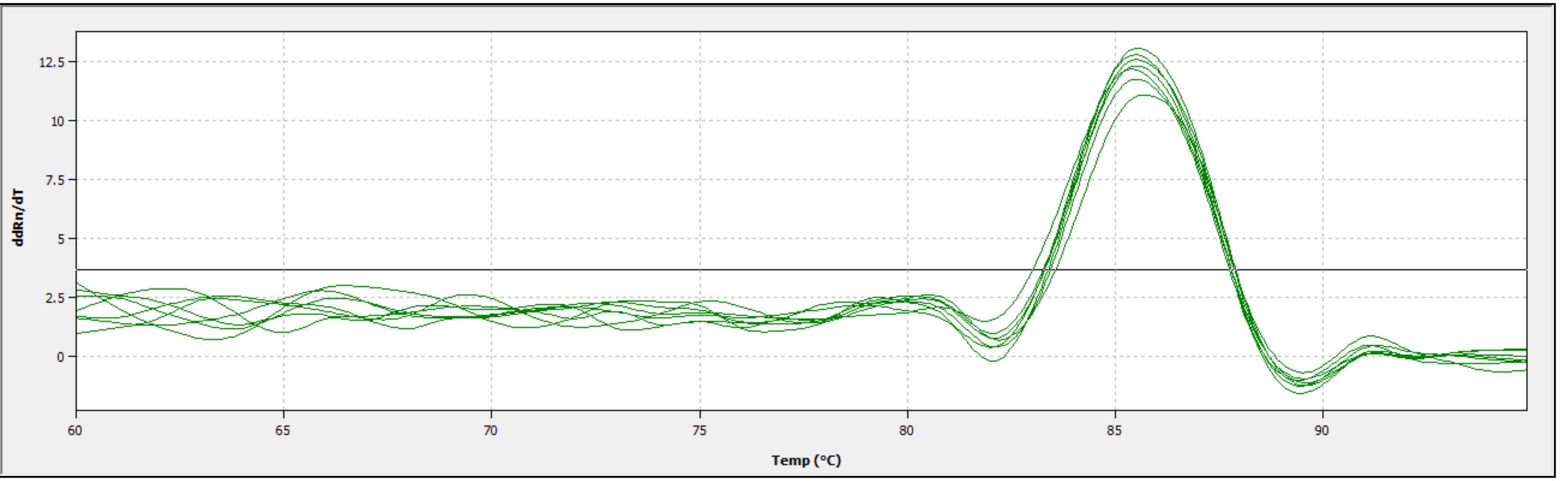

THRAR3

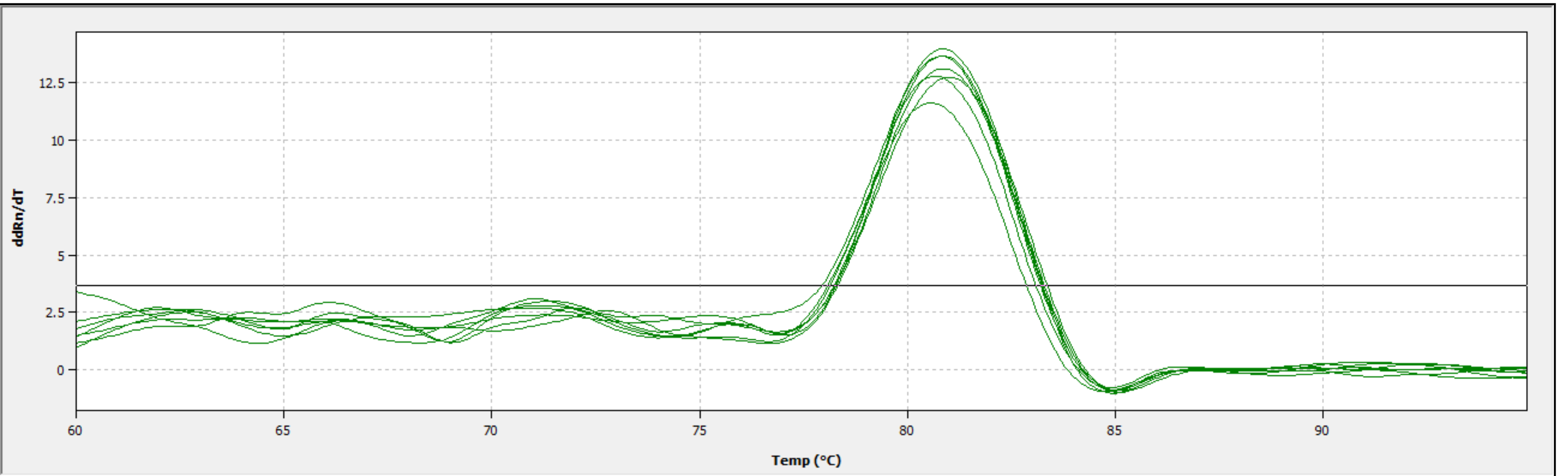

RHOA

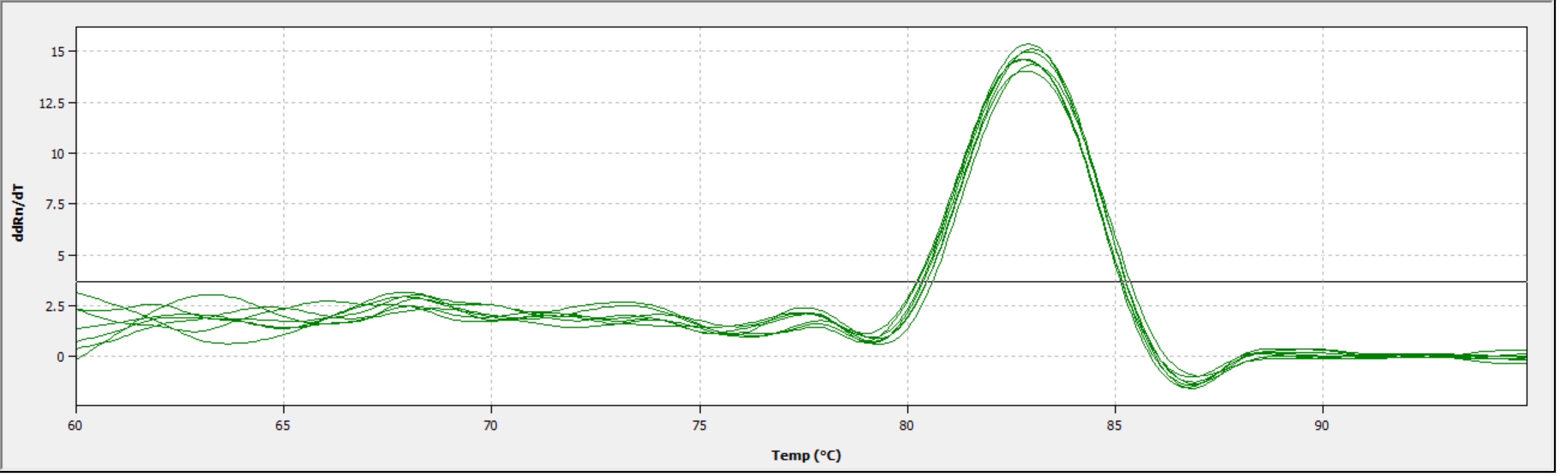

RNF10

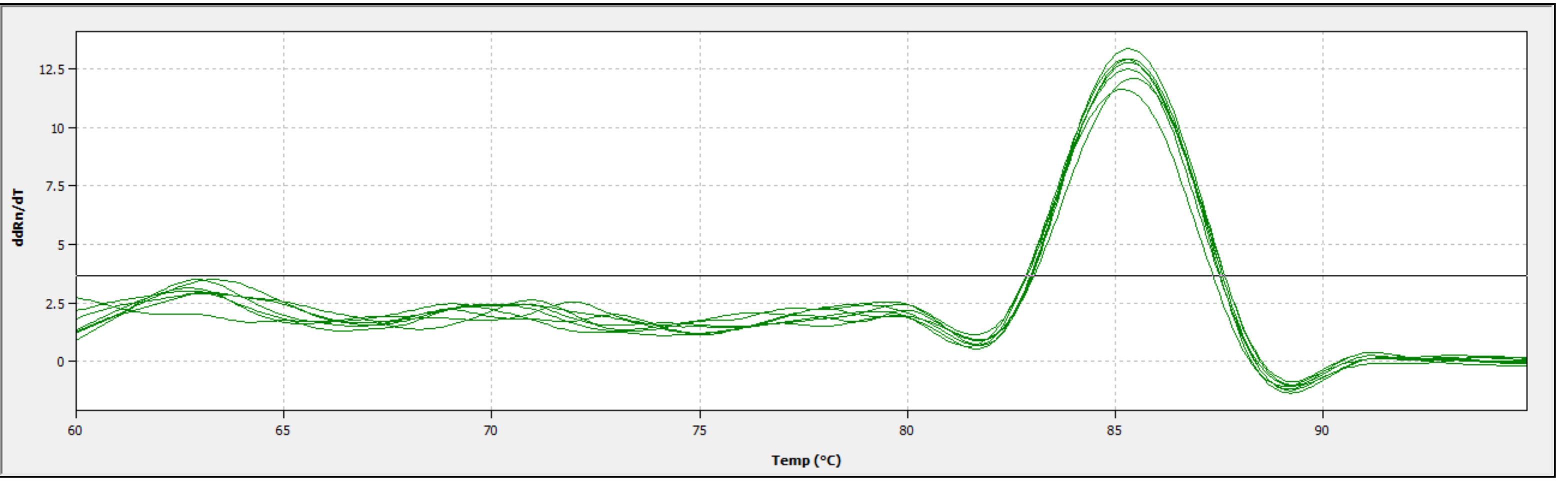

QRICH1

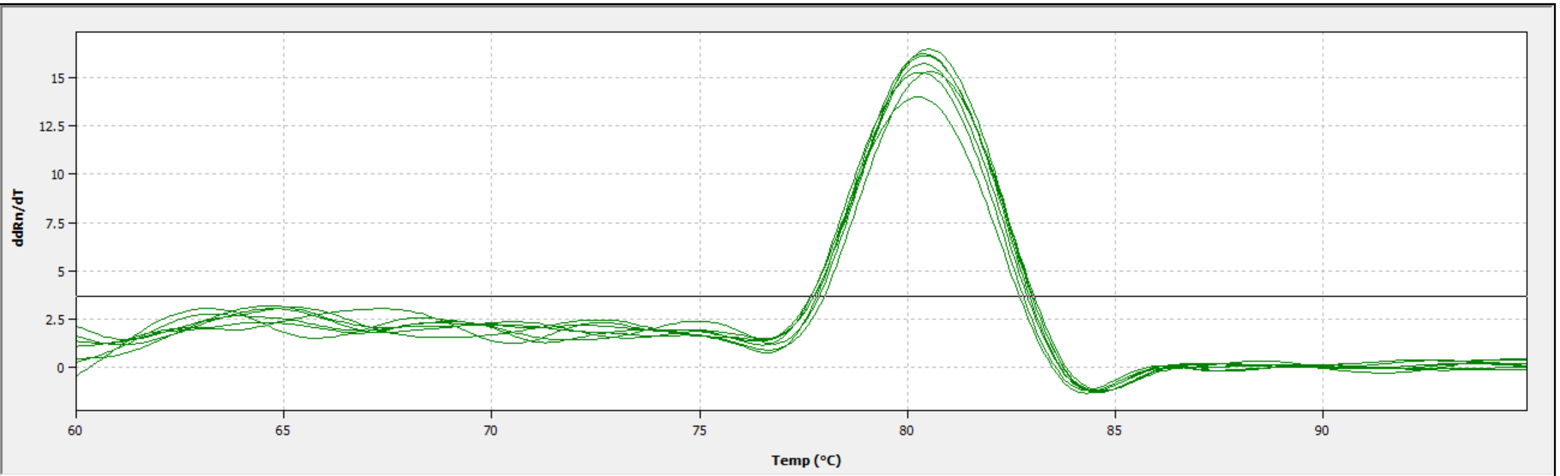

TRA2B

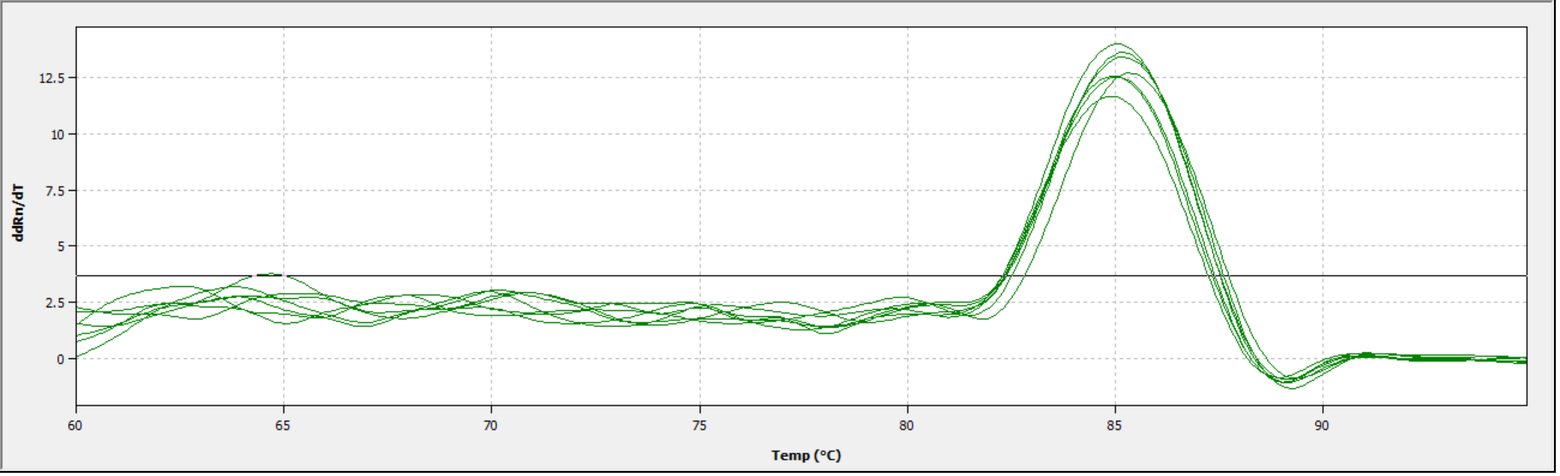

SRSF3

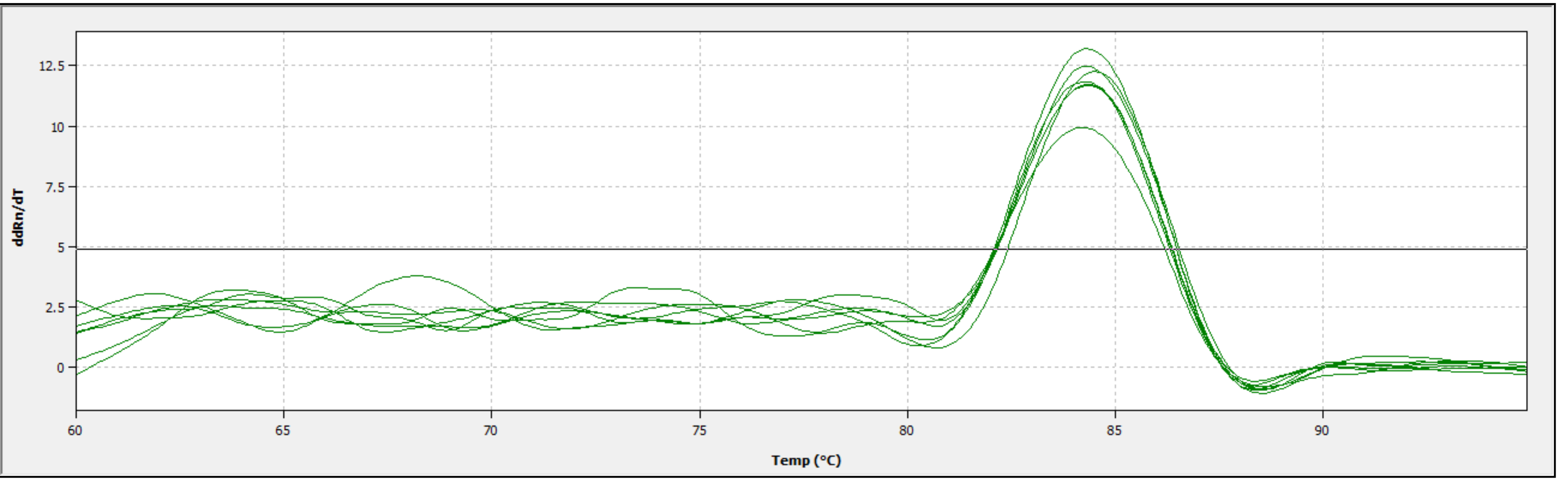

MAPK3

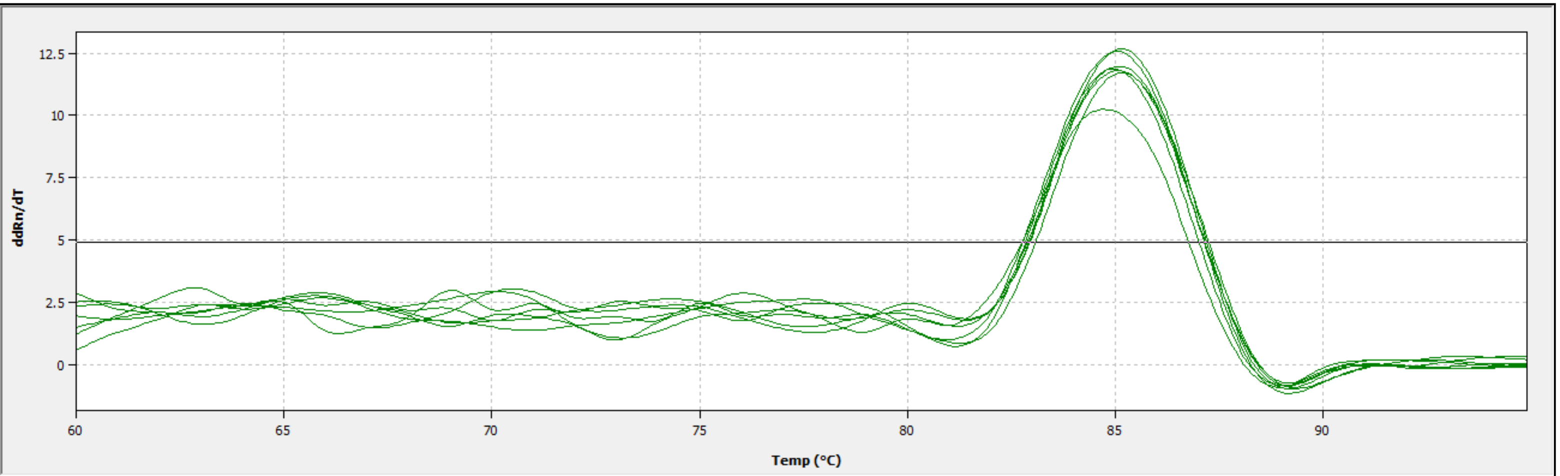

FAAH

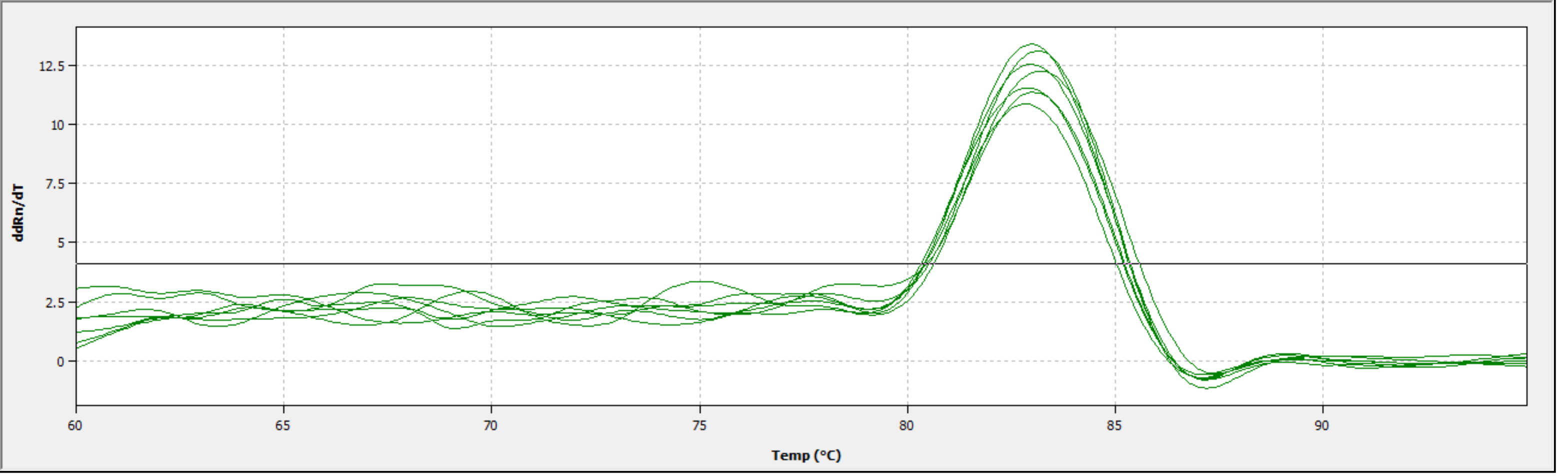
12.5
10
7.5
5
2.5
0
ddRn/dT
60
65
70
75
80
85
90
Temp (°C)

MAPK9

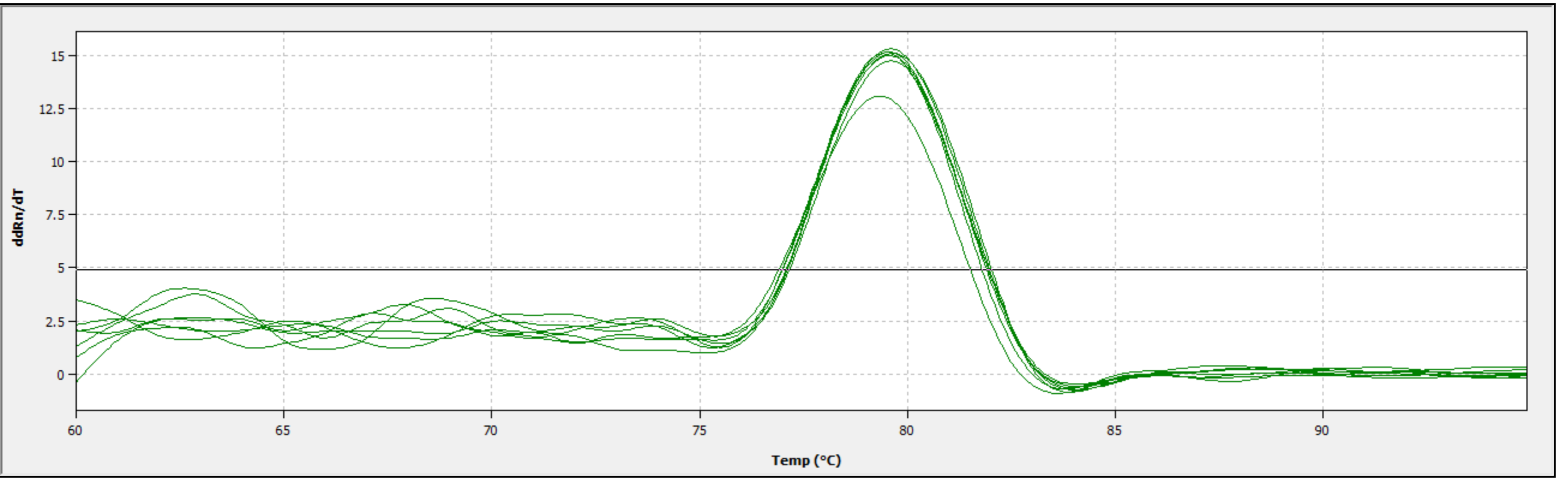
15
12.5
10
7.5
5
2.5
0
ddRn/dT
60
65
70
75
80
85
90
Temp (°C)

HIF1A
