# Supplementary figures and images for: Public transcriptome database-based selection and validation of reliable reference genes for breast cancer research
Source: Biomed Eng Online. 2021 Dec 11;20:124. doi: 10.1186/s12938-021-00963-8 (PMC8665499; doi:10.1186/s12938-021-00963-8)

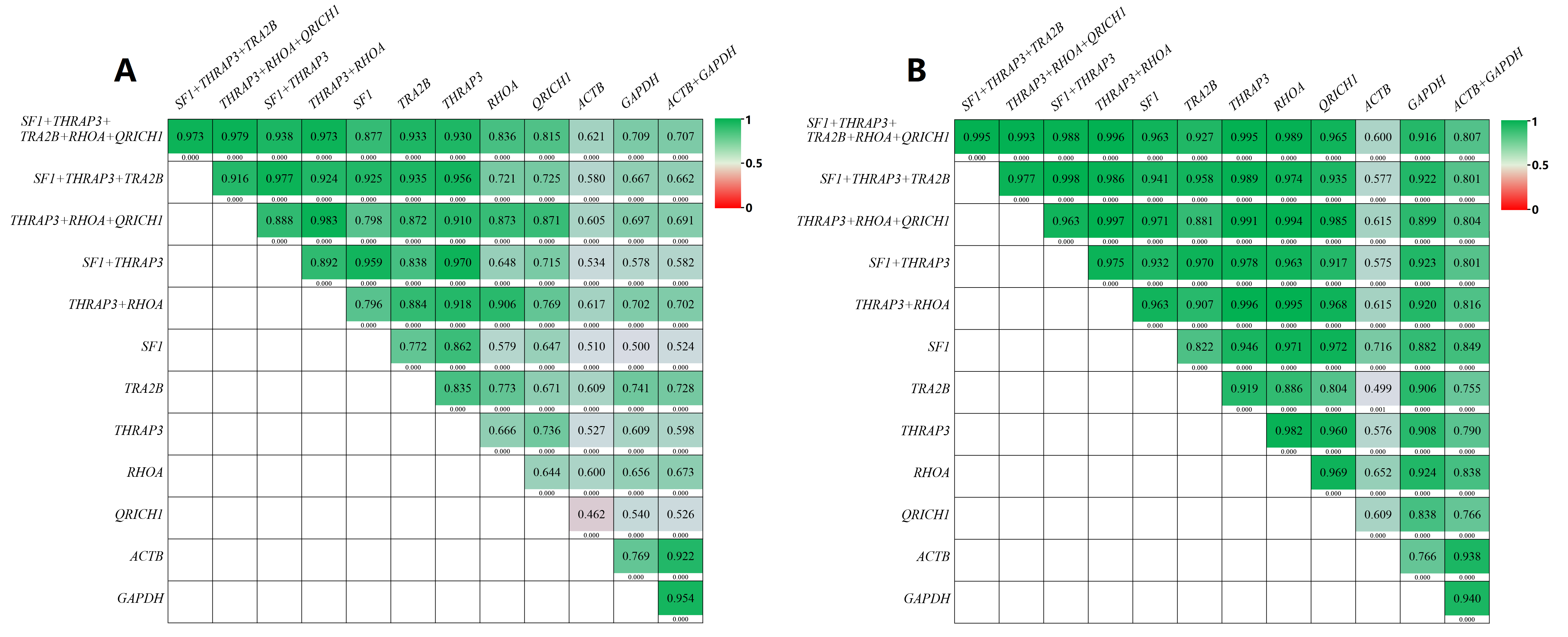

Supplement: Supplementary file 2 — Additional file 2: Figure S2. Heat map of correlation coefficients of relative expression levels based on different normalized RGs. [file 12938_2021_963_MOESM2_ESM.png]
