# Supplementary material for: Public transcriptome database-based selection and validation of reliable reference genes for breast cancer research
Source: Biomed Eng Online. 2021 Dec 11;20:124. doi: 10.1186/s12938-021-00963-8 (PMC8665499; doi:10.1186/s12938-021-00963-8)
Supplement: Supplementary file 3 — Additional file 3: Table S1. The clinical information of all samples in this study. [file 12938_2021_963_MOESM3_ESM.docx]

**Table S1** The clinical information of all samples in this study.

| Subtype | Age | Tumor size（mm） | Pathological type | Stage | Menopausal Status | ER | PR | HER-2 | P53 |
| --- | --- | --- | --- | --- | --- | --- | --- | --- | --- |
| malignant | 43 | 59 | IDC | ⅢA | pre | + | + | - | - |
| malignant | 49 | 30 | IDC | ⅢB | pre | + | + | + | + |
| malignant | 43 | 59 | IDC | ⅢA | pre | + | + | + |  |
| malignant | 48 | 54 | IDC | ⅡB | pre | - | - | - | - |
| malignant | 52 | 35 | IDC | ⅡA | post | - | - | - | + |
| malignant | 48 | 32 | IDC | ⅡB | post | - | - | + | + |
| malignant | 65 | 37 | IDC | ⅢA | post | + | + | - | - |
| malignant | 42 | 34 | IDC | ⅡA | pre | + | + | - | + |
| malignant | 43 | 27 | MA | ⅡA | pre | + | + | - | - |
| malignant | 52 | 34 | IDC | ⅢB | post | - | + | - | - |
| malignant | 47 | 21 | IDC | ⅡA | pre | + | + | - | - |
| malignant | 49 | 15 | MA | ⅡA | pre | + | + | - | - |
| malignant | 41 | 19 | IDC | ⅡB | pre | + | + | + | + |
| malignant | 49 | 43 | DCIS+IDC | ⅡB | pre | + | + | + | + |
| malignant | 49 | 37 | IDC | ⅡA | pre | + | + | + | - |
| malignant | 46 | 38 | IDC | ⅢA | pre | + | + | + | + |
| malignant | 45 | 39 | IDC | ⅡA | pre | + | + | + | + |
| malignant | 46 | 37 | IDC | ⅢB | pre | + | + | + | - |
| malignant | 46 | 50 | IDC | ⅡB | pre | - | - | + | + |
| malignant | 58 | 24 | IDC | ⅡA | post | - | - | + | + |
| malignant | 51 | 39 | IDC | ⅡA | post | - | - | + | - |
| malignant | 55 | 38 | IDC | ⅡA | post | - | - | + | + |
| malignant | 59 | 37 | IDC | ⅡA | post | - | - | + | + |
| malignant | 48 | 41 | IDC | ⅡA | pre | - | - | + | + |
| malignant | 44 | 35 | MBC | ⅡB | pre | - | - | - | + |
| malignant | 47 | 20 | IDC | ⅡA | pre | - | - | - | + |
| malignant | 36 | 30 | IDC | ⅢB | pre | - | - | - | + |
| malignant | 59 | 16 | IDC | ⅡA | post | - | - | - | + |
| malignant | 45 | 29 | IDC | ⅡA | pre | - | - | - | + |
| malignant | 55 | 49 | IDC | IIB | post | - | - | - | - |
| benign | 20 | 21 | BF |  | pre |  |  |  |  |
| benign | 20 | 33 | BF |  | pre |  |  |  |  |
| benign | 20 | 21 | BF |  | pre |  |  |  |  |
| benign | 43 | 17 | BF |  | pre |  |  |  |  |
| benign | 33 | 51 | BF |  | pre |  |  |  |  |
| benign | 46 | 44 | BF |  | pre |  |  |  |  |

[Abbreviation](C:/Users/Administrator/AppData/Local/youdao/dict/Application/8.9.3.0/resultui/html/index.html#/javascript:;):ER: [estrogen](C:/Users/Administrator/AppData/Local/youdao/dict/Application/8.9.3.0/resultui/html/index.html#/javascript:;) [receptor](C:/Users/Administrator/AppData/Local/youdao/dict/Application/8.9.3.0/resultui/html/index.html#/javascript:;); PR: [progesterone](C:/Users/Administrator/AppData/Local/youdao/dict/Application/8.9.3.0/resultui/html/index.html#/javascript:;) [receptor](C:/Users/Administrator/AppData/Local/youdao/dict/Application/8.9.3.0/resultui/html/index.html#/javascript:;); HER-2: human epidermal growth factor receptor 2; IDC, [invasive](C:/Users/Administrator/AppData/Local/youdao/dict/Application/8.9.3.0/resultui/html/index.html#/javascript:;) [ductal](C:/Users/Administrator/AppData/Local/youdao/dict/Application/8.9.3.0/resultui/html/index.html#/javascript:;) [carcinoma](C:/Users/Administrator/AppData/Local/youdao/dict/Application/8.9.3.0/resultui/html/index.html#/javascript:;); MA: [mucinousadenocarcinoma](C:/Users/Administrator/AppData/Local/youdao/dict/Application/8.9.3.0/resultui/html/index.html#/javascript:;); DCIS: ductal carcinoma in situ; MBC: medullary breast carcinoma; BF: breast fibroadenoma.

The stage is based on AJCC 8^th^.

The fist six patiens aquired neoadjuvant chemotherapy, so the stage of tumor is clinical stage.
